# Phosphatidate phosphatase Lipin1 alters mitochondria-associated endoplasmic reticulum membranes (MAMs) homeostasis: effects which contribute to the development of diabetic encephalopathy

**DOI:** 10.1186/s12974-025-03441-3

**Published:** 2025-04-18

**Authors:** Shan Huang, Mengyu Hua, Wei Liu, Ziyun Zhuang, Xiaolin Han, Xiaochen Zhang, Zhonghao Liang, Xiaojing Liu, Nengjun Lou, Shuyan Yu, Shihong Chen, Xianghua Zhuang

**Affiliations:** 1 Department of Endocrinology and Metabolism, The Second Hospital of Shandong University, Jinan, 250033 China; 2 Rehabilitation Hospital, The Second Affiliated Hospital of Shandong University of Traditional Chinese Medicine, Jinan, 250001 China; 3 Department of Endocrinology and Metabolism, The First People’s Hospital of Jinan, Jinan, 250011 China; 4 Department of Clinical Medicine, Heze Medical College, Heze, 274009 China; 5 Multidisciplinary Innovation Center for Nephrology of the Second Hospital of Shandong University, Jinan, 250033 China; 6 Department of Physiology, School of Basic Medical Sciences, Cheeloo College of Medicine, Shandong University, Jinan, 250012 China

**Keywords:** Diabetic encephalopathy, Cognitive dysfunction, Lipin1, MAMs, Mitochondria

## Abstract

Diabetic encephalopathy (DE) is a common, chronic central nervous system complication of diabetes mellitus, and represents a condition without a clear pathogenesis or effective therapy. Findings from recent studies have indicated that a dyshomeostasis of mitochondria-associated endoplasmic reticulum membranes (MAMs) may be involved in the development of neurodegenerative diseases such as DE. MAMs represent a dynamic contact site between mitochondrial and endoplasmic reticulum (ER) membranes, where phospholipid components are exchanged with each other. Previous work within our laboratory has revealed that Lipin1, a critical enzyme related to phospholipid synthesis, is involved in the pathogenesis of DE. Here, we show that Lipin1 is downregulated within the hippocampus of a DE mouse model, an effect which was accompanied with a decrease in MAMs. Knockdown of Lipin1 in the hippocampus of normal mice resulted in a reduction of MAMs, ER stress, abnormal mitochondrial function, as well as impaired synaptic plasticity and cognitive function. These same phenomena were observed in the DE model, while an upregulation of Lipin1 within the hippocampus of DE mice improved these symptoms. Low levels of Lipin1 in DE mice were also associated with neuroinflammation, while an overexpression of Lipin1 significantly ameliorated the neuroinflammation observed in DE mice. In conclusion, Lipin1 ameliorates pathological changes associated with DE in a mouse model via prevention of dyshomeostasis in MAMs. Such findings suggest that Lipin1 may be serve as a new potential target for the treatment of DE.

**Graphical Abstract:**

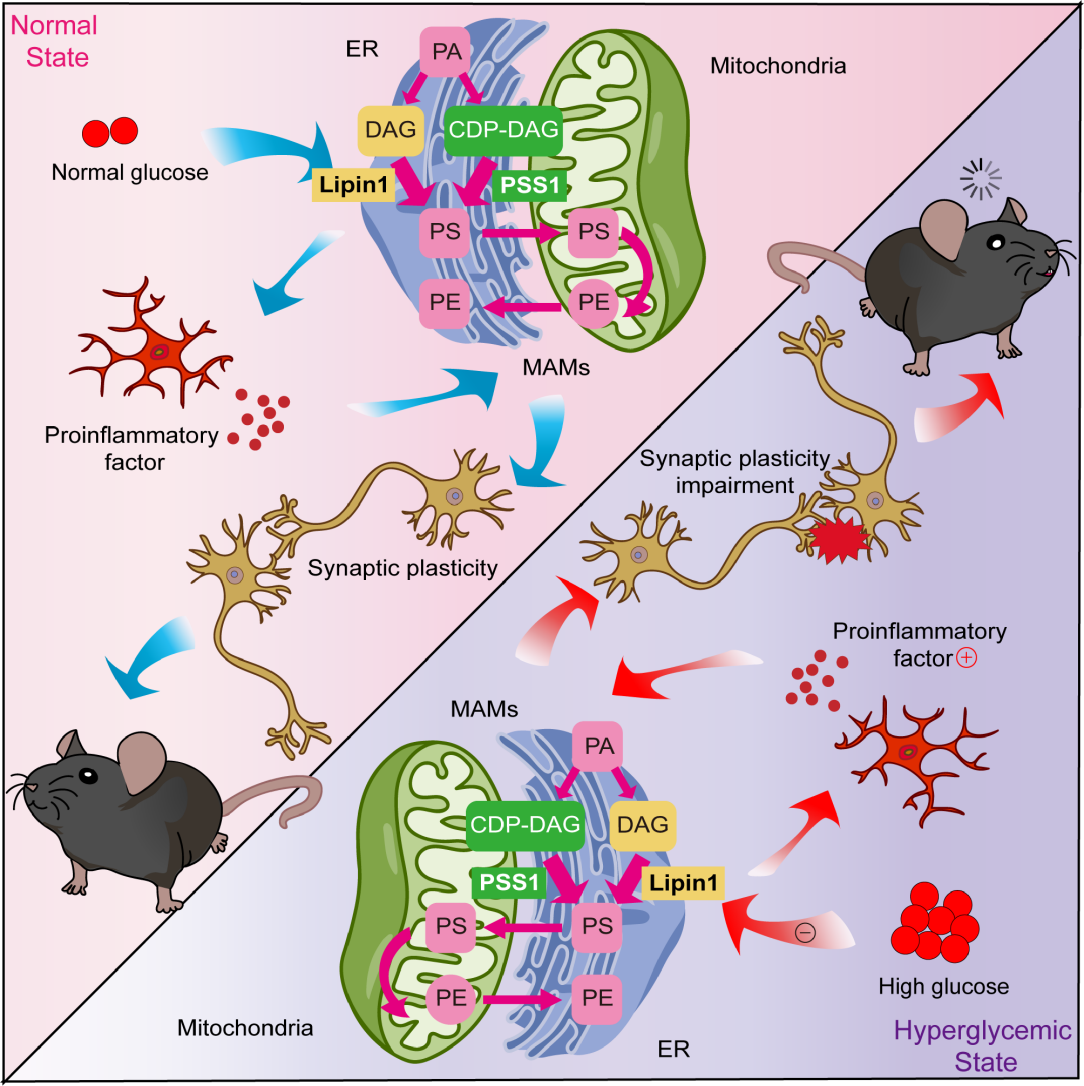

**Supplementary Information:**

The online version contains supplementary material available at 10.1186/s12974-025-03441-3.

## Highlights

Dyshomeostasis of MAMs, which is observed in diabetic encephalopathy, is associated with reduced Lipin1 expression.

A Lipin1 deficiency enhances ER stress, mitochondrial dysfunction and results in cognitive impairment.

An upregulation of Lipin1 suppresses ER stress, improves mitochondrial and, attenuates neuroinflammation and cognitive function in diabetic encephalopathy.

**Supplementary Information:**

The online version contains supplementary material available at 10.1186/s12974-025-03441-3.

## Background

Diabetes mellitus is a common systemic metabolic disease, which is associated with a variety of complications. The cognitive dysfunction, as observed with diabetes, has been receiving increased attention [1]. The concept of diabetic encephalopathy (DE) was introduced in the 1950s as a means to describe this diabetic-induced cognitive dysfunction [2]. With increases in life expectancy of diabetic patients, the prevalence of DE has shown a corresponding increase, which has resulted in serious effects upon these patients’ quality of life. Accordingly, an extreme urgency exists to identify the pathogenesis of DE along with targets for its prevention and treatment.

It has been reported that DE is associated with alterations in synaptic plasticity and neuroinflammation [3], effects which could contribute to the cognitive dysfunction observed, but the exact mechanisms involved remain unclear. Mitochondria represent an important component associated with cognition, as they are involved in energy production, calcium homeostasis, redox reactions, as well as other processes that impact synaptic plasticity [4, 5].

Mitochondrial associated endoplasmic reticulum membranes (MAMs) are dynamic contact regions between the outer mitochondrial membrane and the endoplasmic reticulum (ER) membrane [6]. At this contact site, proteins can interact with each other in the absence of fusion [7]. Thus, functions between these mitochondrial and ER membranes are critically associated with MAMs homeostasis, with a destabilization of this MAMs homeostasis being a common feature of several neurodegenerative diseases, including Alzheimer’s disease [8], Parkinson’s disease [9] and amyotrophic lateral sclerosis [10]. DE is also a neurodegenerative disease and changes in MAMs may also be involved in this condition. For example, within the brains of diabetic mice, there are 144 MAMs-associated proteins showing changes [11]. Moreover, treatment with pharmacologic agents which can modulate MAMs and the composition of their related proteins ameliorate AD-like lesions and the cognitive decline resulting from diabetes [12, 13]. The above findings suggest that abnormal MAMs may be an important pathological component of DE.

As MAMs consist of lipid raft-like structural domains which create a lipid microenvironment [14, 15], any abnormal change in lipid metabolism may lead to MAMs dysfunction. It has been demonstrated that cholesterol and GM1 ganglioside are involved in the formation of MAMs [16, 17]. Interestingly, previous work from our laboratory has demonstrated that Lipin1, a key enzyme in the regulation of phospholipid metabolism, participates in the development of DE. Lipin1 exerts phosphatidate phosphatase (PAP) activity and converts phosphatidate (PA) to diacylglycerol (DAG). And, through its capacity to activate downstream protein kinase C (PKC) and protein kinase D (PKD), Lipin1 can play an important role in the formation of memories [18, 19]. However, whether abnormal phospholipid metabolism induced by Lipin1 alterations causes cognitive dysfunction by affecting MAMs has not been proposed.

An additional damaging factor is inflammation, which is closely linked to MAMs. MAMs are platforms that are necessary for the assembly and activation of inflammasome and participate in inflammatory responses [20, 21]. With inflammation, some proteins are recruited to MAMs [22], however the absence of some proteins associated with MAMs may also cause inflammation [23]. MAMs are involved in the development of diabetic nephropathy and neurodegenerative disease by regulating pathophysiologic processes such as inflammation [24, 25]. Interestingly, Lipin1 has been shown to be involved with inflammation as demonstrated in the liver [26, 27], intestine [28] and muscles [29], but this Lipin1-associated inflammation has yet to be demonstrated within the brain.

Thus, when collating this background information, it seems reasonable to hypothesize that decreased Lipin1 may be involved in inflammatory responses associated with DE via its ability to produce disruptions in MAMs. To test this hypothesis, in this study we constructed both in vivo and in vitro models of DE and altered the expression of Lipin1 in these models. In this way, it was possible to assess whether Lipin1 was associated with the dyshomeostasis of MAMs, inflammation and cognitive damage, which are considered as likely factors responsible for the pathogenesis of DE. Our results indicate that Lipin1 may, in fact, be involved with such effects, suggesting that it may serve as a potential effective target for the treatment of DE.

## Materials and methods

### Animals

Male C56BL/6J mice (7–8 weeks old) were obtained from the Beijing Vital River Laboratory Animal Technology Co., Ltd. (Beijing, China). All mice were housed in a temperature and humidity-controlled environment (12 h/12 h dark/light cycle) with free access to food and water. All experiments involving animals were performed according to the International Guiding Principles for Animal Research as provided by the World Health Organization and approved by the Research Ethics Committee of the Second Hospital of Shandong University.

### In vivo diabetes model

After the mice were fed for one week, a Type 1 diabetes condition was induced in these mice. Mice were fasted for 12–18 h, followed by an intraperitoneal injection of STZ (130 mg/kg) dissolved in citrate buffer. Control mice were injected with an equivalent dose of citrate buffer. On the day of testing, mice were provided with sugar water to prevent hypoglycemia. At 3 days after their STZ injection, a Random Blood Glucose (RBG) sample was taken, with RBG levels of > 16.7mmol/L being considered as the diagnostic criteria for a successful induction of a diabetes model. RBGs and body weights continued to be measured at 1, 2, 4, 8, and 12 weeks after the STZ injection.

### Virus injections

Adeno-associated virus (AAV) was purchased from the Shanghai GeneChem Corporation (Shanghai, China). After anesthesia, the mice were placed in a stereotaxic frame, the head was shaved, the scalp was disinfected and cut to expose the skull. Coordinates for infusions were based on a mouse brain atlas and consisted of -2.00 mm from bregma, ± 1.5 mm medial/lateral and to a depth of -1.5 mm. The Lipin1-overexpressed adeno-associated virus or its blank vector were injected into DE mice (DE + AAV-Lipin1 group and DE + AAV-Ctrl group), while the Lipin1-knockdown adeno-associated virus or its blank vector were injected into normal control mice (WT + AAV-sh Lipin1 group and WT + AAV-Ctrl group). A volume of 1 µl was injected bilaterally into the hippocampus at a flow rate of 0.1 µl/min using a microsyringe. After the injection, the needle remained at the site for 15 min and was then gently removed. The wound was sutured and disinfected and the mice were placed on a warm surface during their recovery.

### Morris water maze (MWM)

The MWM was used to assess spatial learning and memory in these mice. The test was conducted in a circular swimming pool that was evenly divided into 4 quadrants, each with a different eye-catching pattern on the wall. In one quadrant, a circular platform (5 cm in diameter, 15 cm in height) was submerged to a depth of 2 cm below the water surface. On the first day of training the platform was removed and the mice were placed in the water for 1 min to acclimatize to the pool. On the following 5 days, the mice were placed into the water within one of the other 3 non-platform containing quadrants and were permitted 60 s to search for the platform. Mice failing to locate the platform within 60 s were directed to the platform and remained there for 20 s. After 5 days of training, the platform was removed and the mice were placed in the quadrant diagonal to the location of the target quadrant. The total distance traveled and time required to reach the target area and the number of crossings over the target platform area within the 60 s test were recorded. All trials and data were analyzed with use of the software Any-maze (Stoelting Co., Wood Dale, IL, USA).

### Western blot

Mice were euthanized while under anesthesia, the hippocampus removed and stored at -80 ^o^C. Animal tissue and cell proteins were extracted on ice using RIPA lysis buffer (Beyotime, Shanghai, China) supplemented with a protease inhibitor mixture and phenylmethylsulfonyl fluoride (Beyotime, Shanghai, China). The mixture was then cleavedon ice for 30 min, shaking every 10 min. Samples were sonicated and then centrifuged at 12,000 g for 15–20 min at 4 °C. Protein concentrations were measured using the BCA kit (Solarbio, Beijing, China), with SDS-PAGE protein loading buffer (Beyotime, Shanghai, China) then added according to the concentrations measured. Protein samples were separated by sodium dodecyl sulfate polyacrylamide gel electrophoresis and transferred onto PVDF membranes. Samples were then blocked with 5% skim milk for 1 h at room temperature and incubated with primary antibodies, including rabbit anti-BDNF (A1307, ABclonal), rabbit anti-CREB (9197, Cell Signaling Technology), rabbit anti-P-CREB (9198, Cell Signaling Technology), rabbit anti-Lipin1 (A3326, ABclonal), rabbit anti-PSS1 (ab157222, abcam), rabbit anti-CHOP (115204-1-AP, Proteintech), mouse anti-GRP78 (66574-1-lg, Proteintech), rabbit anti-LC3B (ab192890, abcam), rabbit anti-P62 (23214, Cell Signaling Technology), rabbit anti-PINK (23274-1-AP, Proteintech), and rabbit anti-parkin (ab77924, abcam), overnight at 4 °C. On the following day, the membrane was washed 3 times with TBST for 10 min each time, incubated with the secondary antibody conjugated with horseradish peroxidase for 1 h at room temperature, and then washed 3 times with TBST for 10 min each time. An enhanced chemiluminescence detection kit (Merck Millipore, Billerica, MA, USA) was used to assess the images as detected under a chemiluminescence imager (Tanon4800, Shanghai, China). Image J software was used to quantify the intensities of the blots.

### Real‑time quantitative PCR (RT-PCR)

RNA, as isolated from the hippocampus, was extracted using an EZ-press RNA Purification Kit (EZBioscience, Roseville, MN, USA). RNA, isolated from HT22 cells, was extracted using a *SteadyPure* rapid RNA extraction kit (Accurate Biology, Changsha, China). Total RNA (1 µg) was reverse transcribed into cDNA using the *Evo M-MLV* reverse transcription Kit II (Accurate Biology, Changsha, China) according to the manufacturer’s protocol. cDNA was mixed with 2×SYBR Green and primers to form a 10 µl volume preparation. β-actin was used as a housekeeping gene to quantify the mRNA and the data were analyzed using the 2^−ΔΔCT^ method.

### Golgi staining

Mice were anesthetized and euthanized, their brains removed and fixed in 4% PFA for 48 h. The brains were completely submerged in Golgi staining buffer for 48 h, which was changed every 3 days until day 14. Afterwards, brain tissues were immersed in 80% acetic acid, incubated overnight in the dark and then dehydrated in a 30% sucrose solution. After staining, tissue blocks were cut into 100 μm sections, treated with concentrated ammonia and fixing solution, cleaned with xylene and covered with a coverslip. Photographs then captured the dendritic spine densities in the hippocampal region.

### Transmission electron microscope (TEM)

Hippocampal tissue from mice was trimmed into a 1 mm^3^ cube, fixed with 2% glutaraldehyde, dehydrated in increasing gradients of ethanol and embedded. Tissue samples were impregnated with resin and sectioned, then stained with 4% uranyl acetate for 20 min, followed by 0.5% lead citrate on the copper grids. Under observations of the TEM, the portions of the endoplasmic reticulum and mitochondria that were in contact were regarded as MAMs, using the MAMs/mitochondrial perimeter as the parameter to be counted.

### Immunofluorescence

After anesthesia, mice were injected with 0.9% normal saline and 4% paraformaldehyde (PFA). Their brains were removed, fixed with paraformaldehyde for > 24 h, dehydrated using a sucrose gradient, embedded in OCT, and frozen slices of 30 μm were obtained. Frozen sections were washed with PBS, and blocked with BSA for 1 h. The samples were incubated overnight at 4 °C with the appropriate primary antibodies including rabbit anti-Lipin1 (ab181389, abcam), mouse anti-NeuN (66836-1-lg, Proteintech), mouse anti-GFAP (3670, Cell Signaling Technology), goat anti-Iba1 (ab289874, abcam), and rabbit anti-Tom20 (11802-1-AP, Proteintech). Subsequently, the samples were incubated with the fluorescent secondary antibody at room temperature for 1 h and then stained with DAPI (Abcam, Cambridge, UK). Images were captured using a Zeiss LSM800 Laser scanning confocal microscope (Oberkochen, Baden-Wurtberg, Germany).

### Mitochondrial oxidative stress staining

Mouse brain sections were stained with Mito-SOX Red fluorescent dye (5 µM, Invitrogen, California, USA), incubated for 30 min at 37 °C in the dark and nuclei were stained using drops of DAPI (Abcam, Cambridge, UK). Images were captured using a confocal microscope.

### Cell culture treatments

HT22 cells, a mouse hippocampal neuronal cell line, were purchased from Procell Life Science & Technology Co., Ltd (Wuhan, China). Cells were cultivated in HG DMEM medium (Gibco, USA) with 1% penicillin/streptomycin (Biosharp, Hefei, China) and 10% fetal bovine serum (ExCell Bio, Shanghai, China) in a humidified incubator (5% CO2 at 37 ℃). The glucose concentration in HG DMEM medium is 25 mM.

### CCK8 assay

HT22 cells (5 × 10^3^) were seeded in 96-well plates and cultured until the cells adhered to the wall. The cells were then treated with high-glucose DMEM medium containing 2% FBS and 1% penicillin/streptomycin for 6 h and then replaced with DMEM medium containing 25, 50, 75, 100, 125 or 150 mM glucose or mannitol for 24–48 h. After 2–3 h of incubation with the CCK8 reagent (MCE, Shanghai, China), cell activity was determined using a microplate reader at a wavelength of 450 nm.

### In vitro diabetes model

HT22 cells were seeded in culture dishes and cultured until the cells adhered to the wall. Cells were cultured for 48 h using a high-glucose DMEM medium (Gibco, USA) with a glucose concentration of 100 mM or a high-glucose DMEM medium (Gibco, USA) with a glucose concentration of 25 mM.

### Virus infection

HT22 cells were seeded into 96-well plates to establish a suitable multiplicity of infection (MOI). The cells were then seeded into 24-well plates for formal infection and screened with puromycin. Lentivirus (LV) was purchased from the Shanghai GeneChem Corporation (Shanghai, China). Cells infected with Lipin1 overexpressing lentivirus or their blank vectors were cultivated with 100 mM DMEM medium (100G + LV-Lipin1 group and 100G + LV-Ctrl group), while cells infected with the Lipin1 knockdown lentivirus or their blank vectors were cultivated with 25 mM DMEM medium (25G + LV-sh Lipin1 group and 25G + LV-Ctrl group) for 48 h.

### Fluorescent colocalization

HT22 cells treated with 25 mM or 100 mM glucose medium for 48 h were stained with ER-tracker (Solarbio, Beijing, China) and Mito-tracker (Invitrogen, California, USA), respectively. Hoechst33342 staining was performed, with images being obtained using a Zeiss LSM800 Laser scanning confocal microscope (Oberkochen, Baden-Wurtberg, Germany).

### Statistical analysis

All statistical analyses were performed using GraphPad Prism 9.5.1 and presented as means ± SEMs. Independent group Student’s t-tests (two-tailed) were used for comparisons involving two independent groups. One-way analyses of variance (ANOVAs) were used for comparisons involving the four groups, with the Tukey test used for post-hoc pairwise comparisons. A *p* < 0.05 was required for results to be considered as statistically significant.

## Results

### Mice show cognitive dysfunctions at 12 weeks after STZ-induced diabetes

**Fig. 1:**
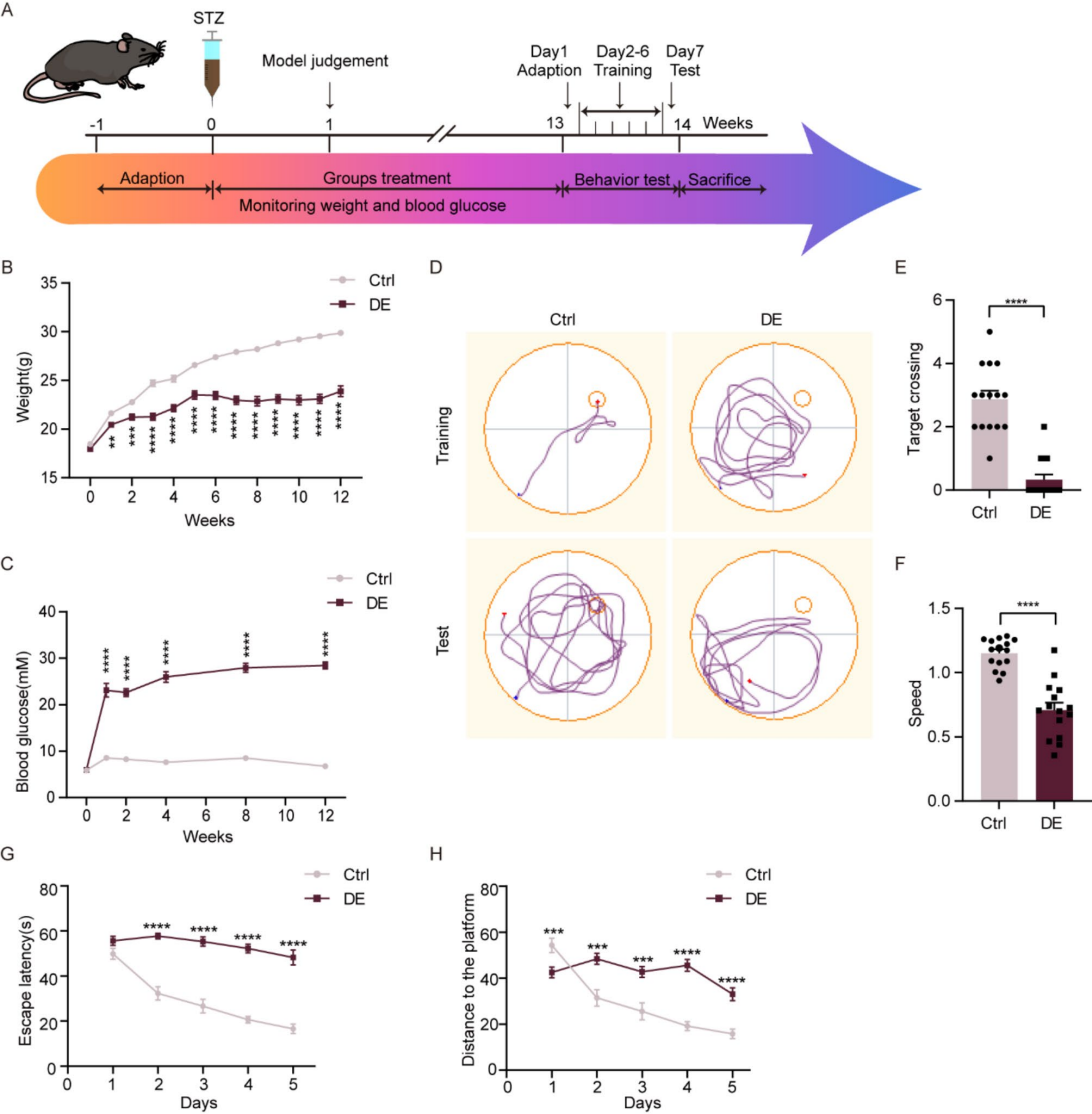
Mice show cognitive dysfunctions which model diabetic encephalopathy (DE) at 12 weeks after STZ injection. (**A**) Experimental paradigms for establishing the DE mouse model and behavioral experiments in DE and control mice. (**B**) Body weights and (**C**) Blood glucose levels at 0–12 weeks after STZ/control injections (*n* = 15 per group). (**D**) Movement trajectories in the Morris water maze. (**E**) Number of times mice in each group crossed the target platform during the test period (*n* = 15 per group). (**F**) Swimming speeds in the Morris water maze (*n* = 15 per group). (**G**) Escape latencies during the training period (*n* = 15 per group). (**H**) Distance traveled to the target platform during the training period (*n* = 15 per group). All data are shown as means ± SEMs. **p < 0.01, ***p < 0.001, and ****p < 0.0001, Ctrl vs. DE. Ctrl, Control. DE, Diabetic Encephalopathy

The procedure for establishing the diabetic mouse model is summarized in Fig. 1A. At 1 week post-STZ injection, these mice had significantly lower body weights (*p* < 0.01) and significantly higher blood glucose levels (*p* < 0.0001) as compared with mice in the control group. The blood glucose values of ≥ 16.7 mmol/L in these STZ-treated mice fulfilled our criteria for diabetes. Moreover, these mice showed obvious symptoms of diabetes consisting of polydipsia, polyphagia and polyuria, providing further evidence indicating a successful induction of a diabetic model (Fig. 1B-C, Additional file 1: Fig. S1). Over time, mice in the diabetic group continued to gain less weight and had higher levels of blood glucose than that observed in the control mice.

As based on results obtained with the Morris water maze, at 12 weeks after STZ treatment, these mice showed clear evidence of cognitive dysfunctions which modeled diabetic encephalopathy (DE). In specific, these DE mice traversed the target platform area significantly less than control mice (*p* < 0.0001) (Fig. 1D-E) and showed escape latencies that were significantly longer than that of control mice beginning with the second day of the training period (*p* < 0.0001) (Fig. 1G). In addition, swimming speeds of DE mice were lower than that of control mice (*p* < 0.0001) (Fig. 1F), with further analyses of these swimming speeds revealing that DE mice swam longer distances to reach the target platform than the control group (*p* < 0.001) (Fig. 1H). And, the effect of speed was also excluded by analysis of covariance (Additional file 2: Table. S1-S7).

### Lipin1 expression levels, MAMs and indicators of cognitive function are decreased in the DE mouse model

**Fig. 2:**
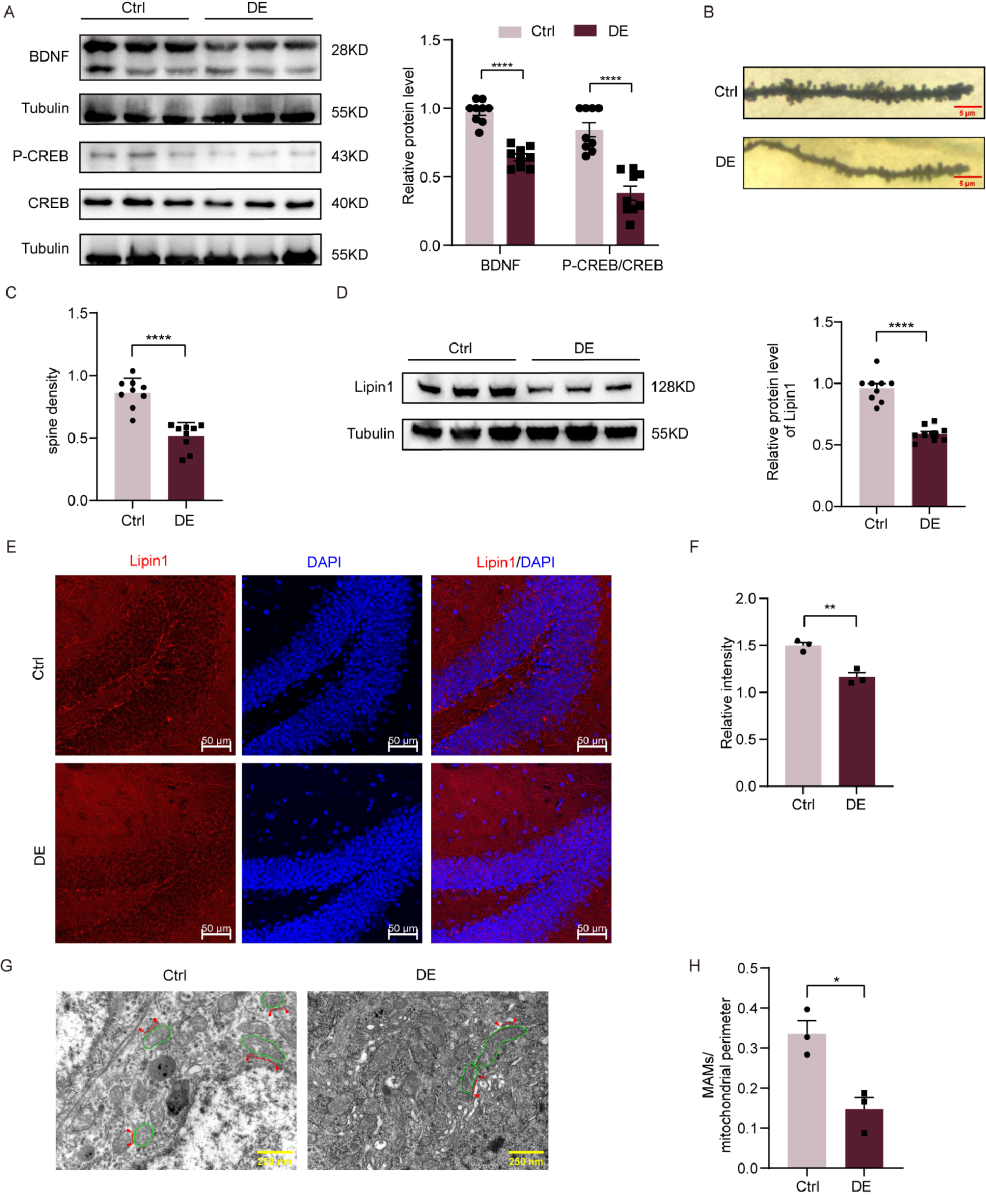
Decreased Lipin1 expression levels, MAMs and indicators of cognitive function in the DE mouse model. (**A**) Representative Western blot images showing relative protein levels of BDNF and CREB phosphorylation in DE and control mice (*n* = 9 per group). (**B**) Representative images of hippocampal dendritic densities. Scale bar is 10 μm. (**C**) Number of dendrites in hippocampal neurons (*n* = 9 per group). (**D**) Representative Western blot images of Lipin1 (*n* = 9 per group) (**E**) Representative immunofluorescent images of Lipin1. Scale bar is 50 μm. (**F**) Representative immunofluorescent images showing relative intensities of Lipin1 (*n* = 3 per group). (**G**) Representative images from transmission electron microscopy showing MAMs and mitochondria. Red, MAMs. Green, mitochondria. Scale bar is 250 nm. (**H**) Ratio of MAMs to mitochondrial perimeters as observed in electron microscopy images. All data are shown as means ± SEMs. *p < 0.05, **p < 0.01 and ****p < 0.0001, Ctrl vs. DE. Ctrl, Control. DE, Diabetic Encephalopathy

In DE mice, hippocampal levels of BDNF expression (*p* < 0.0001) and CREB phosphorylation (*p* < 0.0001) were decreased as compared with control mice (Fig. 2A). Golgi staining was used to observe any potential changes in the number of dendritic spines and morphological structure of the hippocampus. Compared with that of control mice, dendritic spine densities within DE mice were significantly reduced (*p* < 0.0001) and the morphology was obviously atrophied (Fig. 2B-C). The changes obtained in these parameters are consistent with the cognitive dysfunction observed in these DE mice.

Results from our Western blot assays showed that the expression levels of Lipin1 in the hippocampus of DE mice were significantly lower than that in the control group (*p* < 0.0001) (Fig. 2D). Similarly, immunofluorescent staining revealed that the fluorescent intensities of Lipin1 in the hippocampus of DE mice were significantly lower than that of the control group (*p* < 0.01) (Fig. 2E-F). Such findings indicate that hippocampal levels of Lipin1 in DE mice are lower than that observed in normal mice. Lipin1 was co-localized with neurons, astrocytes, and microglia respectively. The results showed that in DE mice, lipin1 was significantly reduced in neurons, while there was no significant difference in the changes of lipin1 in glial cells, suggesting that Lipin1 reduction occurs mainly in neurons in DE. (Additional file 3: Fig. S2). When assessing the ultrastructure of the hippocampus with use of transmission electron microscopy, we found that the ratios of MAMs located adjacent to the mitochondrial perimeter were significantly lower in the DE versus control group (*p* < 0.05) (Fig. 2G-H), indicating that MAMs were reduced and disorganized in these DE mice.

### Lipin1 expression and MAMs are decreased in neuronal cells treated with high glucose levels

**Fig. 3:**
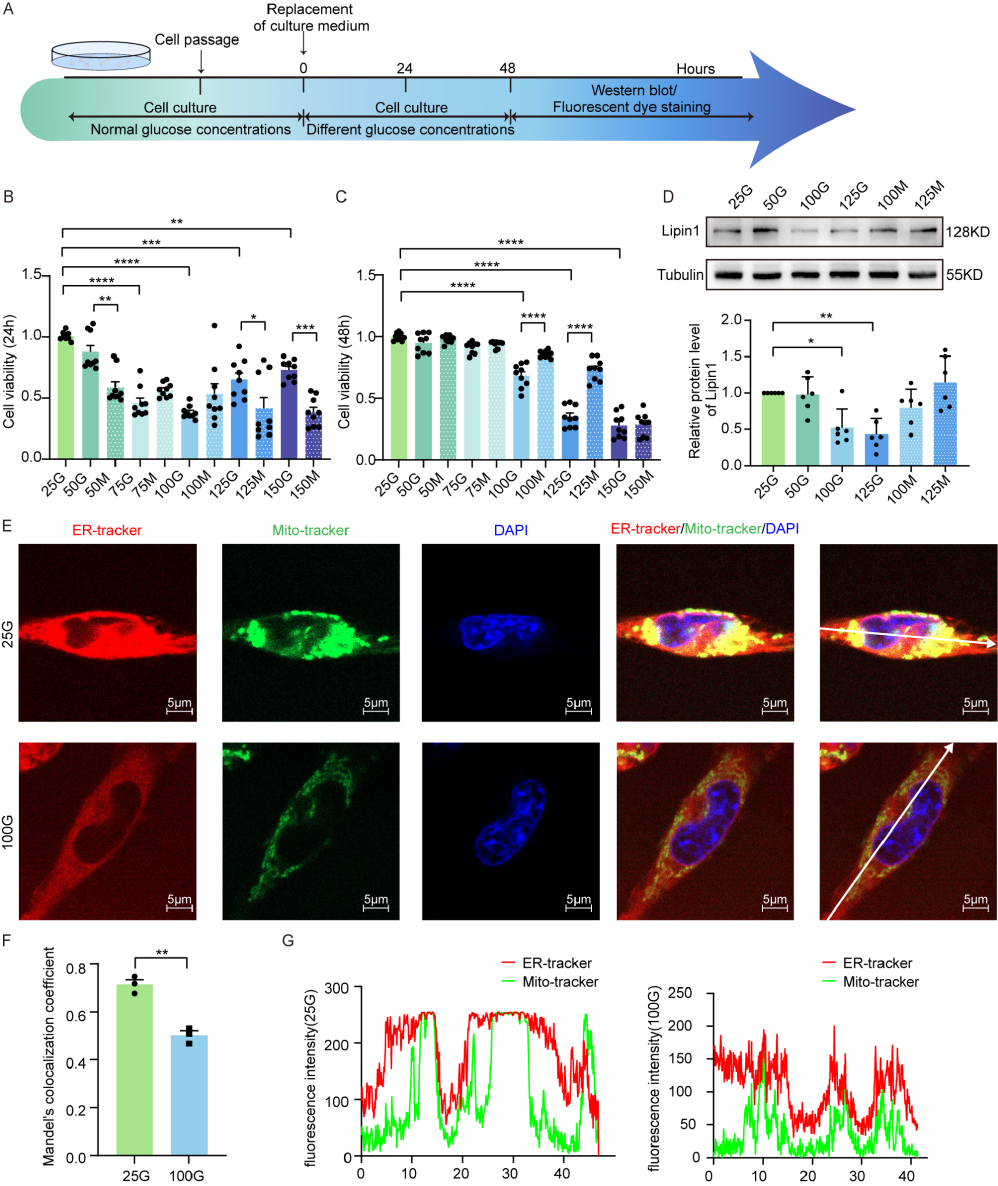
High glucose levels alter the state of HT22 neuronal cells. (**A**) Experimental paradigms for the HT22 cell experiments. Cell viability after (**B**) 24 h and (**C**) 48 h of treatment with glucose and mannitol (*n* = 9 per group). (**D**) Representative Western blot images showing relative protein expressions of Lipin1 in cells from the treatment groups described above (*n* = 6 per group). (**E**) Fluorescent images showing co-localizations of endoplasmic reticulum and mitochondria in cells. Scale bar is 5 μm. (**F**) Ratios of co-localized MAMs to mitochondrial regions in fluorescent images were analysed by Manders’ Coefficients (*n* = 3 per group). (**G**) Intensity of endoplasmic reticulum and mitochondria fluorescence at the white vector in cells. All data are shown as means ± SEMs. *p < 0.05, **p < 0.01, ***p < 0.001, and ****p < 0.0001, 25G vs. 100G OR other groups. G, Glucose. M, Mannitol. For example, 25G, 25 mM Glucose. 50 M, 50 mM Mannitol

The general design of this experiment is illustrated in the schematic diagram of Fig. 3A. HT22 cells were treated with different concentrations of glucose and mannitol for 24 h and 48 h, respectively, and cell activity was assessed using a CCK8 assay. The activity observed in HT22 cells treated with glucose for 48 h showed a gradual decrease as a function of increasing glucose concentrations, with statistically significant differences between 100 mM glucose versus the mannitol treatment groups (*p* < 0.0001). These results suggest that the effect of high glucose on cell activity was greater than that of the osmolality with this concentration (Fig. 3B-C). Therefore, a 100 mM glucose concentration was chosen as a condition to simulate the high glucose environment of diabetes in subsequent experiments with these cells.

Expression levels of Lipin1 in HT22 cells were significantly decreased in response to exposures of 100 or 125 mM glucose (*p* < 0.05, *p* < 0.01), indicating that high glucose concentrations led to a decrease in Lipin1 expression in these neuronal cells (Fig. 3D). To assess whether a co-localization exists between the endoplasmic reticulum and mitochondria in HT22 cells ER-tracker and Mito-tracker were used. More MAMs were obtained in HT22 cells treated with 25 versus 100 mM glucose concentrations (*p* < 0.01) suggesting that high levels of glucose produced a decrease and homeostatic imbalance in MAMs (Fig. 3E-G).

The mitochondrial function in neurons was detected after high glucose treatment. Compared with neurons treated with normal glucose concentration, after treatment with 100 mM glucose concentration, the mitochondrial membrane potential decreased and oxidative stress was enhanced (Additional file 4: Fig. S3A-D). In addition, the length of mitochondria in neurons was shorter after high glucose treatment (Additional file 4: Fig. S3E-F).

### Changes in Lipin1 alter cognitive function in mice

**Fig. 4:**
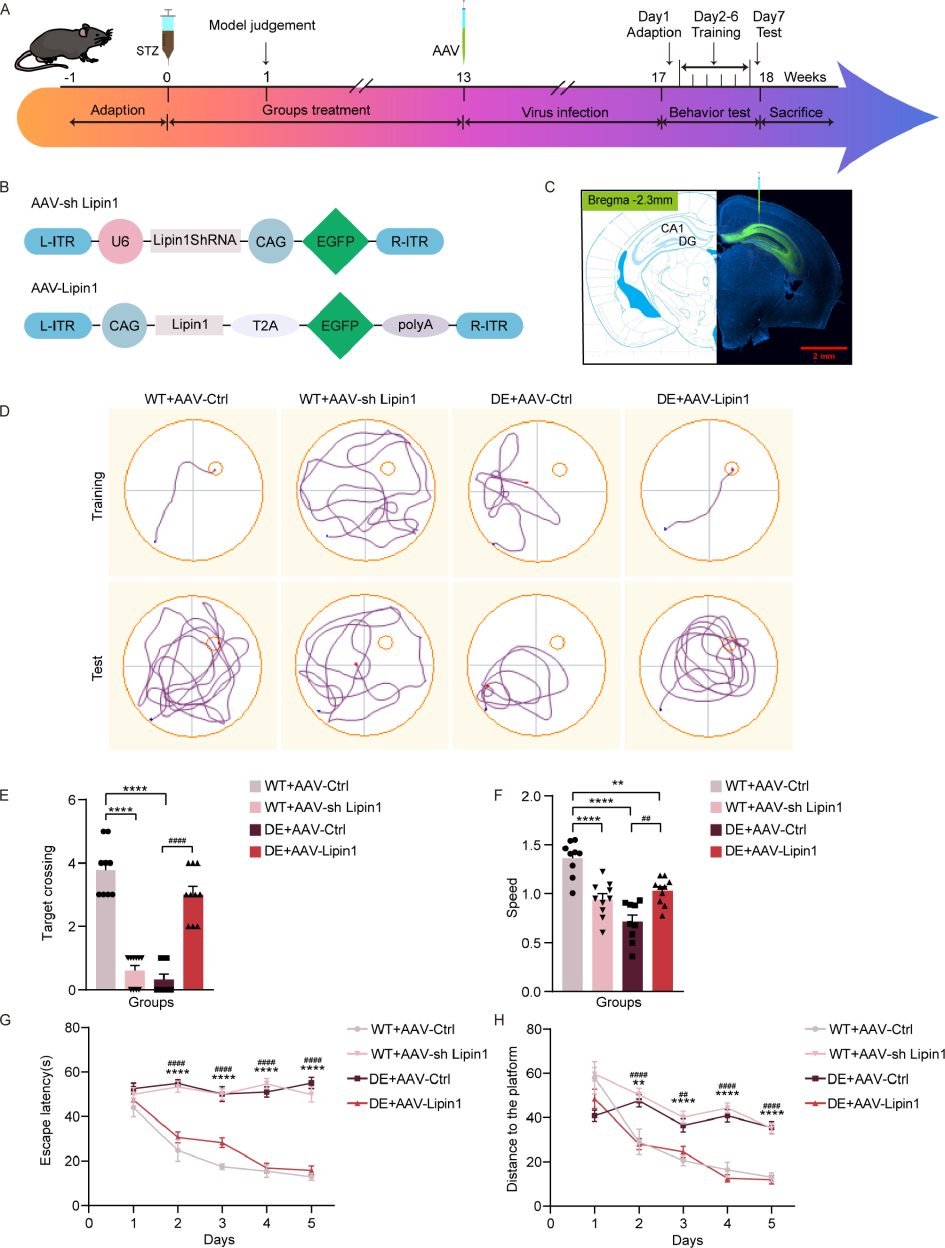
Changes in Lipin1 alter cognitive function in mice. (**A**) Experimental paradigms for stereotactic injections into the hippocampus and behavioral experiments. (**B**) Schematics of AAV vectors engineered to knock-down and overexpress Lipin1. (**C**) Demonstration of an adeno-associated virus injection into the hippocampus. Scale bar is 2 mm. (**D**) Movement trajectories of the four groups of mice in the Morris water maze. (**E**) Number of crossings of the target platform during the test period (*n* = 9–10 per group). (**F**) Swimming speeds in the Morris water maze (*n* = 9–10 per group). (**G**) Escape latencies during the training period (*n* = 9–10 per group). (**H**) Distance traveled to reach the target platform during the training period (*n* = 9–10 per group). All data are shown as means ± SEMs. **p < 0.01 and ****p < 0.0001, WT + AAV-Ctrl vs. Other groups. ^##^*p* < 0.01 and ^####^*p* < 0.0001, DE + AAV-Lipin1 vs. Other groups

DE mice were injected with an overexpression of Lipin1 adeno-associated virus or the blank vector, while control mice were injected with an interfering Lipin1 expression adeno-associated virus or its blank vector (Fig. 4A-B). Viral infections were verified by section scanning and virally regulated Lipin1 expression levels were examined using real‑time quantitative PCR and Western blot. DE mice injected with an overexpressing Lipin1 adeno-associated virus showed increased levels of Lipin1 transcription (*p* < 0.001) and translation (*p* < 0.0001). Transcription (*p* < 0.0001) and translation (*p* < 0.001) levels of Lipin1 were significantly reduced in the normal group of mice injected with an interfering Lipin1 adeno-associated virus (Fig. 4C, Additional file 5: Fig. S4).

Morris water maze tests, as conducted at four weeks after stereotactic injection of the adeno-associated virus, revealed that DE mice with an overexpression of Lipin1 crossed the target platform more times than DE mice receiving the blank virus (*p* < 0.0001). Control mice injected with the virus that interferes with Lipin1 expression (*p* < 0.0001) and DE mice receiving a blank virus (*p* < 0.0001) crossed the target platform significantly less than that of control mice with the blank virus (Fig. 4D-E). During the training period, DE mice with an overexpression of Lipin1 showed significantly decreased escape latencies versus DE mice receiving the blank virus mice (*p* < 0.0001). Control mice with an interfered Lipin1 expression had significantly increased escape latencies compared to those receiving the blank virus (*p* < 0.0001) (Fig. 4G). To exclude the effect of the swimming speed as contributing to these effects (*p* < 0.01, *p* < 0.0001) (Fig. 4F), the distance traveled by each group to reach the target platform was analyzed. Beginning with the second day of the training period, DE mice with an overexpression of Lipin1 virus showed significantly shorter distances traveled to the target platform than that of DE mice injected with the blank virus (*p* < 0.01, *p* < 0.0001). In control mice with an interfered Lipin1 expression distances traveled to reach the target platform were significantly increased as compared with control mice injected with the blank virus (*p* < 0.01, *p* < 0.0001) (Fig. 4H). In addition, the effect of speed was also excluded by analysis of covariance (Additional file 6: Table. S8-S12).

### Changes in Lipin1 affect indicators of cognitive function and MAMs

**Fig. 5:**
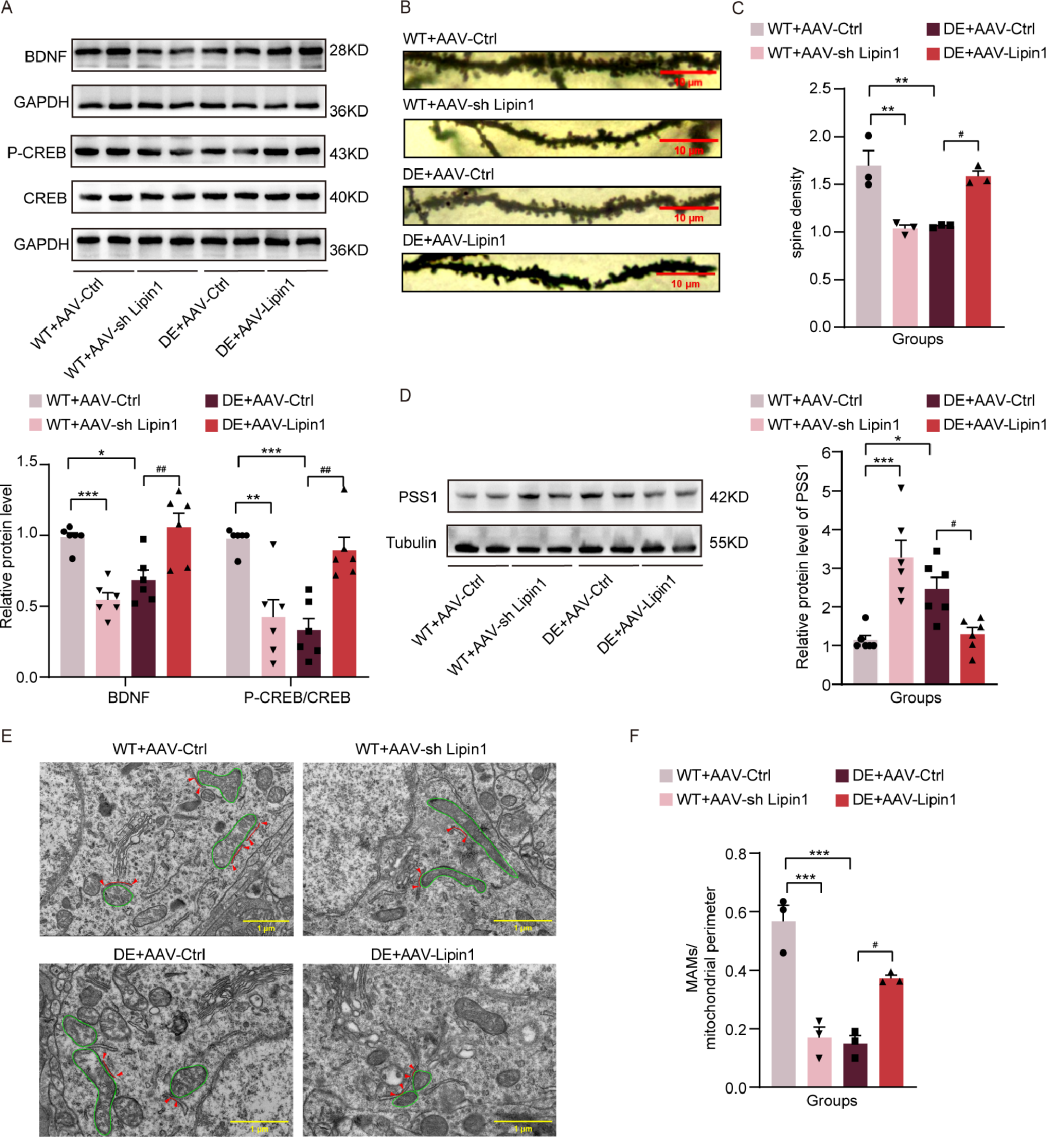
Changes in Lipin1 affect indicators of cognitive function and MAMs. (**A**) Representative Western blot images showing relative protein levels of BDNF and CREB phosphorylation in mice with altered levels of Lipin1 expression (*n* = 6 per group). (**B**) Representative images of hippocampal dendritic spine densities in mice. Scale bar is 10 μm. (**C**) Hippocampal spine densities vary as a function of alterations in Lipin1 expression (*n* = 3 per group). (**D**) Representative Western blot images showing relative protein expression levels of PSS1 (*n* = 6 per group). (**E**) Representative transmission electron microscopy images showing MAMs and mitochondria. Red, MAMs. Green, mitochondria. Scale bar is 1 μm. (**F**) Ratio of MAMs to mitochondrial perimeter in electron microscopy images (*n* = 3 per group). All data are shown as means ± SEMs. *p < 0.05, **p < 0.01, and ***p < 0.001, WT + AAV-Ctrl vs. Other groups. ^#^*p* < 0.05, and ^##^*p* < 0.01, DE + AAV-Lipin1 vs. Other groups

Hippocampal levels of BDNF (*p* < 0.001) and CREB phosphorylation (*p* < 0.01) in control mice injected with an interfering Lipin1 expression virus were significantly decreased as compared with that of control mice injected with the blank virus. Levels of BDNF (*p* < 0.05) and CREB phosphorylation (*p* < 0.001) in DE mice receiving the blank virus were significantly decreased as compared with control mice injected with the blank virus. Hippocampal levels of BDNF (*p* < 0.01) and CREB phosphorylation (*p* < 0.01) in DE mice overexpressing Lipin1 were significantly increased compared with DE mice receiving the blank virus (Fig. 5A). Golgi staining was used to assess potential changes in dendritic spine number, morphology and structure within the hippocampus of mice with altered Lipin1 levels. In mice receiving the blank virus, dendritic spine densities were significantly reduced in DE versus control mice (*p* < 0.01). When comparing the hippocampal dendritic spine densities of control mice injected with the blank versus interfering Lipin1-expressing virus, the latter group not only showed significant reductions in densities (*p* < 0.01) but also substantial morphologic atrophy. In DE mice injected with the overexpressing Lipin1 virus, there was a significant increase in dendritic spine densities (*p* < 0.05) and an improved hippocampal morphology as compared with DE mice receiving the blank virus (Fig. 5B-C). The above results suggest that overexpression of Lipin1 enhances factors which can be associated with an improved cognitive function in DE mice, while an Lipin1 deficiency diminishes the activity of these factors which can then lead to development of the cognitive deficits observed in these DE mice.

When visualizing MAMs with use of TEM in mice receiving the blank virus, DE mice showed a significantly reduced MAM/mitochondrial perimeter as compared with that observed in control mice (*p* < 0.001). In control mice injected with the interfering Lipin1 virus there was a significant decrease in the ratio of MAM/mitochondrial perimeter compared with control mice injected with the empty virus (*p* < 0.001). This ratio of MAM/mitochondrial perimeter was increased in DE mice overexpressing Lipin1 compared with DE mice injected with the empty virus (*p* < 0.05). Such findings suggest that an overexpression of Lipin1 ameliorates the reduction of MAMs as induced by diabetes (Fig. 5E-F). We also measured the expression levels of phosphatidylserine synthetase 1 (PSS1), a protein in the region of MAMs. Our Western blot results showed that PSS1 expression was elevated in control mice receiving the interfering Lipin1-expressing virus (*p* < 0.001) and in DE mice with a blank virus (*p* < 0.05) as compared with control mice injected with the blank virus. In DE mice overexpressing Lipin1, PSS1 expression levels were similar to that of control mice carrying a blank virus (*p* < 0.05) (Fig. 5D).

### Changes in Lipin1 affect Endoplasmic reticulum and mitochondrial function

**Fig. 6:**
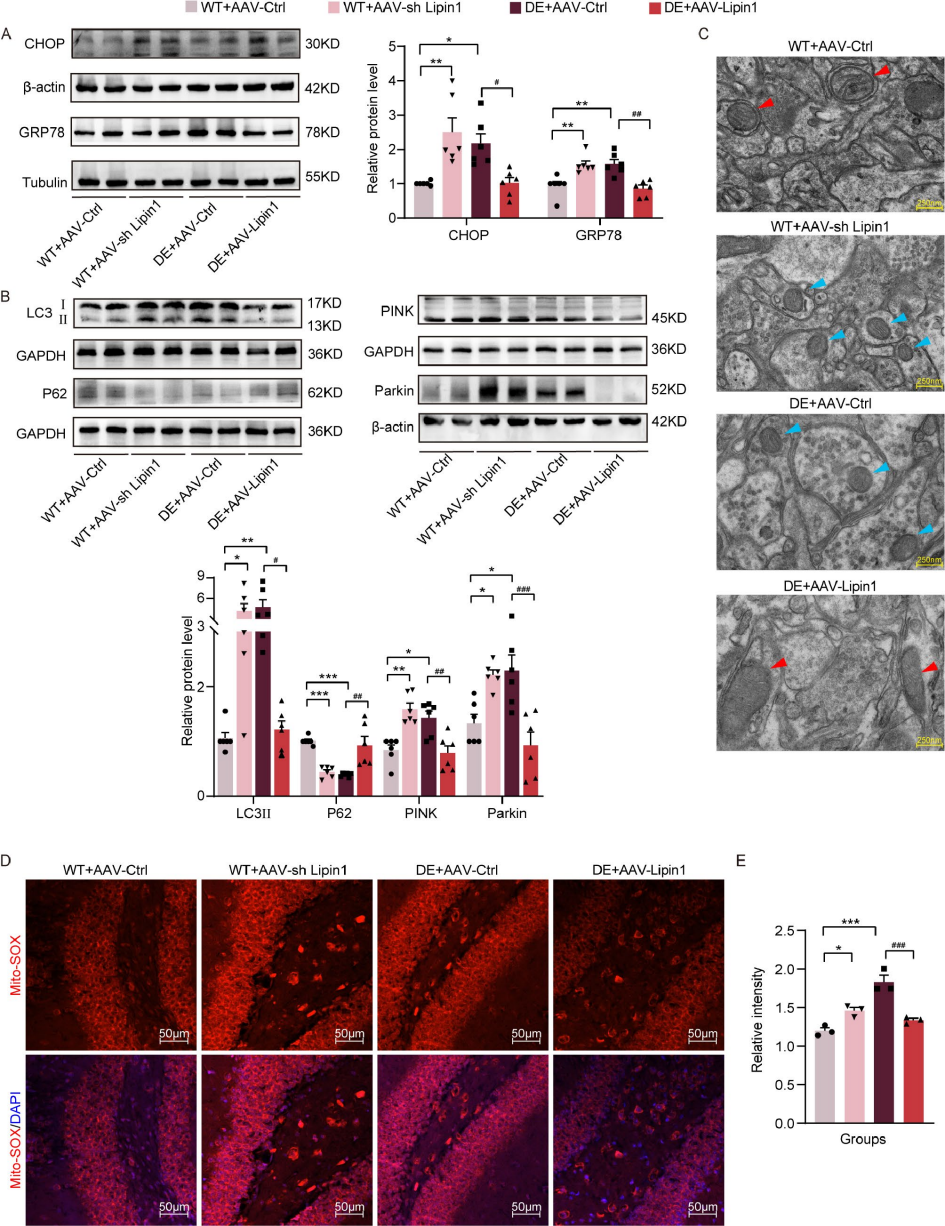
Changes in Lipin1 affect endoplasmic reticulum and mitochondrial function. (**A**) Representative Western blot images showing relative protein levels of CHOP and GRP78 (*n* = 6 per group). (**B**) Representative Western blot images showing relative protein levels of LC3II, P62, PINK and Parkin (*n* = 6 per group). (**C**) Electron microscopy images of mitochondrial morphology. Red arrows, mitochondria with regular morphology and clear cristae. Bule arrows, mitochondria with swollen, disordered or disappeared cristae. (**D**) Representative immunofluorescent images of Mito-SOX. Scale bar is 50 μm. (E) Relative levels of oxidative stress. All data are shown as means ± SEMs. *p < 0.05, **p < 0.01, and ***p < 0.001, WT + AAV-Ctrl vs. Other groups. ^#^*p* < 0.05, ^##^*p* < 0.01, and ^###^*p* < 0.001, DE + AAV-Lipin1 vs. Other groups

Endoplasmic reticulum stress levels were increased in control mice with an interfering Lipin1-expressing virus and in DE mice with an empty virus, as based on the elevated protein expressions of C/EBP-homologous protein (CHOP) (*p* < 0.01, *p* < 0.05) and 78-kD glucose-regulated protein (GRP78) (*p* < 0.01, *p* < 0.01) observed in these mice versus control mice carrying a blank virus. In the DE mouse group overexpressing Lipin1 there were reduced protein expression levels of CHOP (*p* < 0.05) and GRP78 (*p* < 0.01) as compared with DE mice receiving the empty virus, suggesting a reduced level of endoplasmic reticulum stress in the former group (Fig. 6A). When compared with the control group receiving an empty virus, controls receiving the interfering Lipin1 virus and DE mice with an empty virus showed significant increases in LC3II (*p* < 0.05, *p* < 0.01), PTEN Induced putative Kinase 1 (PINK) (*p* < 0.01, *p* < 0.05), and Parkin (*p* < 0.05, *p* < 0.05) and a significant decrease in P62 (*p* < 0.001, *p* < 0.001). These results suggest that the level of mitophagy is increased under conditions of a Lipin1 deficit. In DE mice with an overexpression of Lipin1 there was a decrease in LC3II (*p* < 0.05), PINK (*p* < 0.01), Parkin (*p* < 0.001) and an increase in P62 (*p* < 0.01) as compared with DE mice receiving a blank virus, suggesting that the level of mitophagy was decreased by Lipin1 overexpression (Fig. 6B).

When assessing mitochondrial morphology with use of TEM we observed a full and regular appearance, with cristae that were well defined in control mice with an empty virus. In contrast, morphology of mitochondria in DE mice with an empty virus and in the control mouse group with an interfered Lipin1 expressing virus demonstrated a swollen mitochondrial morphology that was disorganized and blurred and contained broken cristae, effects which were substantially improved within DE mice overexpressing Lipin1 (Fig. 6C). Mito-SOX was also determined as a means to assess mitochondrial levels of oxidative stress. We found that, in contrast to that observed in control mice with a blank virus, DE mice with an empty virus (*p* < 0.001) and control mice with an interfering Lipin1-expressing virus (*p* < 0.05) showed significant increases in Mito-SOX levels, indicating an increased degree of oxidative stress in these two groups. However, in DE mice overexpressing Lipin1 there was a decrease in the level of oxidative stress (*p* < 0.001) (Fig. 6D). In addition, co-staining with Tom20 and MitoSOX was performed to exclude the influence of mitochondrial number on the results. The results also demonstrated that knockdown of Lipin1 and diabetes led to an increase in mitochondrial oxidative stress, and the oxidative stress in diabetic mice was improved after Lipin1 overexpression (Additional file 7: Fig. S5A-B).

### Changes in Lipin1 affect inflammation-related indicators

**Fig. 7:**
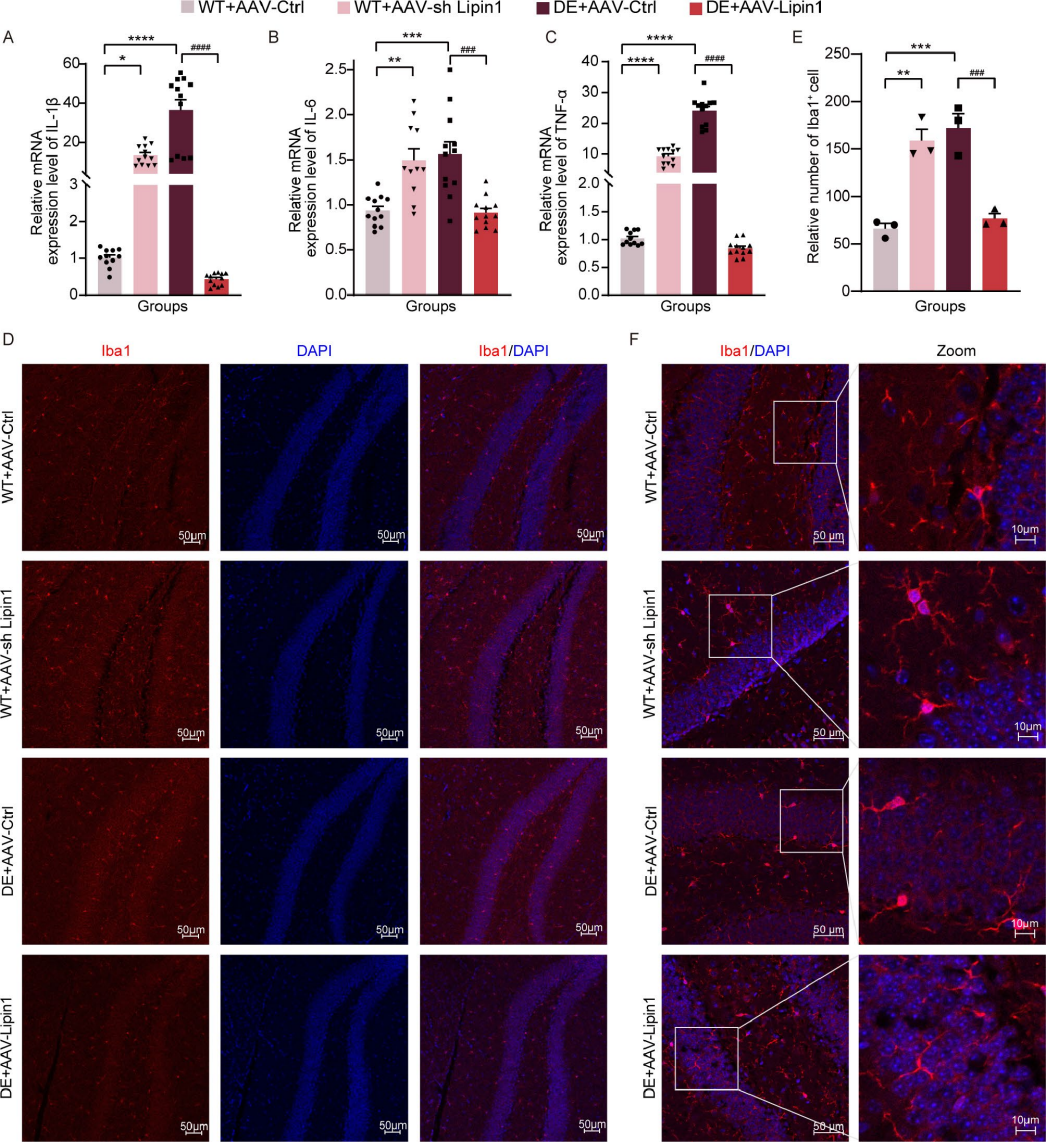
Changes in Lipin1 affect inflammation-related indicators. RT-PCR assays of mRNA expression levels of (**A**) IL-β, (**B**) IL-6 and (**C**) TNF-α (*n* = 11–12 per group). (**D**) Representative immunofluorescent images of Iba1^+^ cells. Scale bar is 50 μm. (**E**) Relative number of Iba1^+^ cells (*n* = 3 per group). (**F**) Morphology of Iba1^+^ cells. Scale bar is 50 μm. All data are shown as means ± SEMs. **p < 0.01, ***p < 0.001, and ****p < 0.0001, WT + AAV-Ctrl vs. Other groups. ^##^*p* < 0.01, ^###^*p* < 0.001, and ^####^*p* < 0.0001, DE + AAV-Lipin1 vs. Other groups

Expression levels of three pro-inflammatory factors, IL-β, IL-6, and TNF-α, were significantly increased in DE mice injected with the blank virus (*p* < 0.0001, *p* < 0.001, *p* < 0.0001) and control mice injected with the interfering Lipin1virus (*p* < 0.05, *p* < 0.01, *p* < 0.0001) as determined using RT-PCR. In DE mice injected with a virus that overexpressed Lipin1, expressions of IL-β, IL-6, and TNF-α were significantly decreased as compared with that of DE mice receiving a blank virus (*p* < 0.0001, *p* < 0.001, *p* < 0.0001) (Fig. 7A-C).

Microglia are innate immune cells of the central nervous system and are major mediators of neuroinflammation. As Iba1 is specifically expressed in microglia, it has been used as a marker for microglia. Therefore, in the next series of experiments Iba1 was labeled using immunofluorescent staining and levels of these Iba1^+^ cells were analyzed. Significantly more Iba1^+^ cells were observed in DE mice infected with the blank virus (*p* < 0.001) and in control mice infected with the virus that interferes with Lipin1 expression (*p* < 0.01) as compared with that in control mice infected with the blank virus. With an overexpression of Lipin1 in DE mice the number of Iba1^+^ cells were significantly reduced (*p* < 0.001) (Fig. 7D-E). With regard to microglia, we found that in DE mice with an empty virus and control mice with an interfered Lipin1 expressing virus there was an activation of these microglia with a large volume of cytosol and few branches. In contrast, microglia in DE mice overexpressing Lipin1, exhibited a resting state with a small volume of cytosol and many branches, an appearance which was similar to that of control mice injected with a blank virus (Fig. 7F).

### Changes in Lipin1 affect MAMs, Endoplasmic reticulum and mitochondria in HT22 cells

**Fig. 8:**
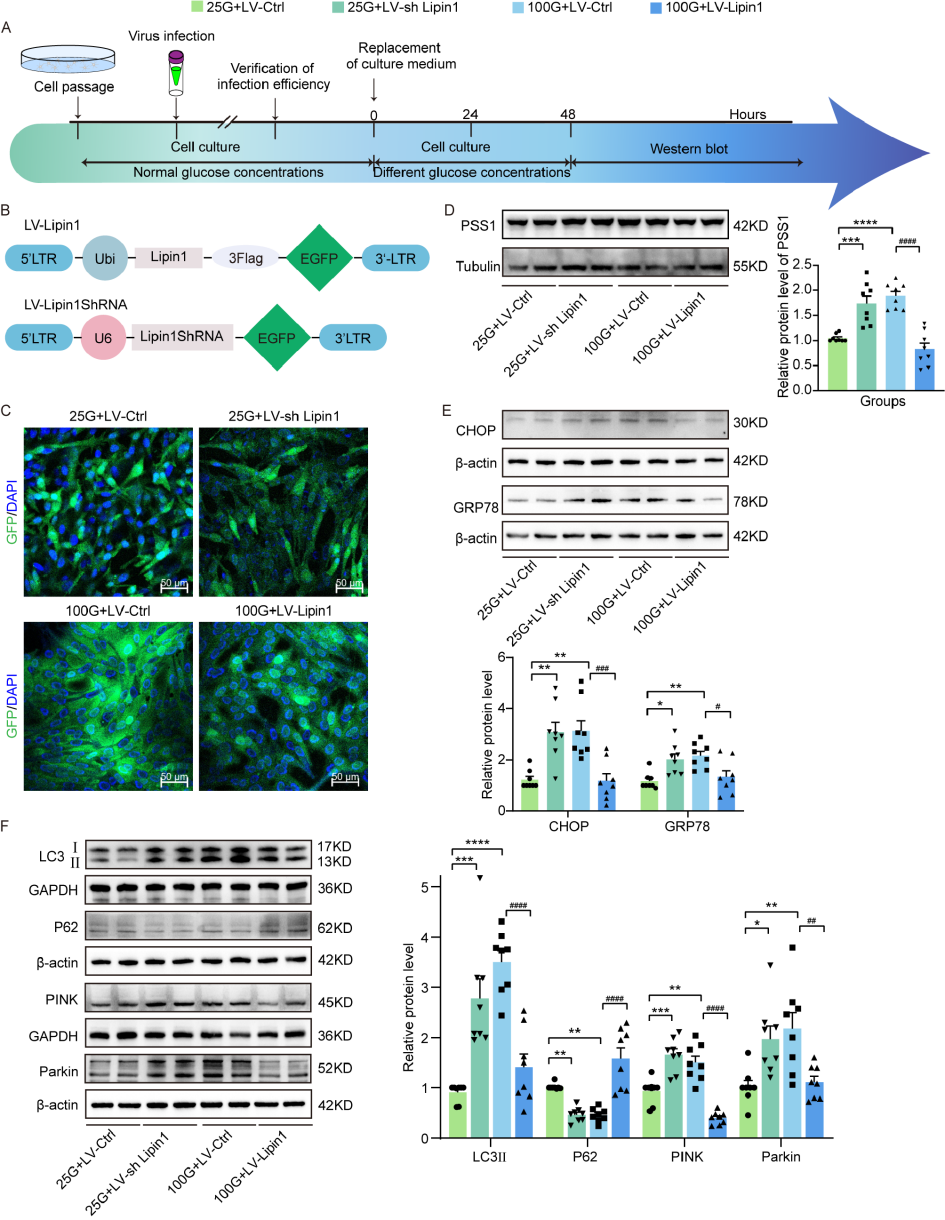
Changes in Lipin1 affect MAMs, endoplasmic reticulum and mitochondria in HT22 cells. (**A**) Experimental paradigms for the cell culture experiments. (**B**) Schematics of LV vectors engineered to knock-down or overexpress Lipin1. (**C**) Representative images of HT22 cells infected with the viruses. Representative Western blot images showing relative protein expressions after LV injection in HT22 cells for (**D**) PSSI, (**E**) CHOP and GRP78 and (**F**) LC3II, P62, PINK and Parkin (*n* = 8 per group). All data are shown as means ± SEMs. *p < 0.05, **p < 0.01, ***p < 0.001, and ****p < 0.0001, 25G + LV-Ctrl vs. Other groups. ^#^*p* < 0.05, ^##^*p* < 0.01, ^###^*p* < 0.001, and ^####^*p* < 0.0001, DE + AAV-Lipin1 vs. Other groups

HT22 cells were infected with lentivirus to either overexpress or interfere with Lipin1 expression, with their respective blank viral infections serving as controls (Fig. 8A-B). The observation of green fluorescence verified their infection, while their transcriptional and translational levels were determined with use of RT-PCR and WB (Fig. 8C, Additional file 8: Fig. S6). The 100mM DMEM medium was used to simulate the diabetic state and the 25mM DMEM medium to simulate the normal/control state.

PSS1 expression was increased in the 100 mM DMEM medium-treated blank virus group and the 25 mM DMEM medium-treated cells with an interfered Lipin1 expression as compared with the 25 mM DMEM medium-treated cells group with an empty virus (*p* < 0.001, *p* < 0.0001). These findings suggest that a high glucose state or a reduced Lipin1 expression can result in disordered MAMs. In cells overexpressing Lipin1 and cultured in 100 mM DMEM medium, there was a significant decrease in PSS1 expression (*p* < 0.0001), an effect which was similar to that as observed in the normal state, and suggests that this increased expression of Lipin1 diminished the glucose-induced destabilization of MAMs (Fig. 8D). The 100 mM DMEM medium-treated group receiving virus-empty cells and the 25 mM DMEM medium-treated group with an interference in the expression of Lipin1 showed significant increases in protein expression levels of the endoplasmic reticulum stress-related indicators CHOP (*p* < 0.01, *p* < 0.01) and GRP78 (*p* < 0.01, *p* < 0.05) as compared to that in the 25 mM DMEM medium-treated group with virus-empty cells. Protein expression levels of CHOP (*p* < 0.001) and GRP78 (*p* < 0.05) were significantly decreased in the group of cells overexpressing Lipin1 and cultured with 100 mM DMEM medium as compared with the group of empty viral cells treated with the 100 mM DMEM medium, suggesting that endoplasmic reticulum stress was reduced with an overexpression of Lipin1 (Fig. 8E). In 25 mM DMEM medium-treated cells with an interfered Lipin1 expression and the 100 mM DMEM medium-treated cells with a blank virus LC3II (*p* < 0.001, *p* < 0.0001), PINK (*p* < 0.001, *p* < 0.01) and Parkin (*p* < 0.05, *p* < 0.01) were significantly increased and P62 significantly decreased (*p* < 0.01, *p* < 0.01) as compared with that observed in 25 mM DMEM medium-treated cells with a blank virus. These results suggest that high concentrations of glucose or low expression levels of Lipin1 lead to increased levels of mitophagy. In cells infected with an empty virus and cultured in 100 mM DMEM medium, LC3II (*p* < 0.0001), PINK (*p* < 0.0001), and Parkin (*p* < 0.01) were significantly decreased and P62 (*p* < 0.0001) significantly increased when compared with cells overexpressing Lipin1 and cultured in 100 mM DMEM medium. These findings indicate that Lipin1 produced a decrease in mitophagy as induced by high glucose levels (Fig. 8F).

## Discussion

Diabetes mellitus is a metabolic disease characterized by chronic hyperglycemia, a condition whose exact pathogenesis remains unclear. It can involve multiple organs, among which complications involving the central nervous system have received increasing attention. According to epidemiological evidence, patients with both type 1 (T1DM) and type 2 (T2DM) diabetes mellitus can exhibit varying degrees of mild to moderate cognitive decline [30]. In this study, we found that in a mouse model of diabetic encephalopathy (DE) high levels of glucose resulted in reductions of phosphatidate phosphatase Lipin1 and a disorganization of MAMs within the hippocampus. Such effects alter ER and mitochondrial function, impair synaptic plasticity, and eventually produce neuroinflammation leading to cognitive impairment.

Results from a previous study within our laboratory indicated that a cognitive dysfunction was present in a T1DM mouse model as observed at 12 weeks after treatment with STZ [31, 32]. In this study, changes in the number and morphology of dendritic spines in DE model can reflect alterations in cognitive functions [33–35]. Other notable indicators of cognitive functioning, including the expression of BDNF and phosphorylation levels of CREB, were also reduced in the DE model [36, 37].

A current view is that normal activity of endoplasmic reticulum and mitochondria are important for the maintenance of cognitive function, with any dysfunction in these organelles resulting in neuronal senescence as well as age-associated cognitive declines [38, 39]. Notably, mitochondria and endoplasmic reticulum, as important subcellular organelles regulating cellular material and energy metabolism, are not independent and communicate through MAMs to maintain normal functions, and a homeostatic imbalance of MAMs is an important pathological feature of several neurodegenerative diseases [40]. However, whether such mechanisms may be involved in the pathogenesis of DE is currently unclear. Therefore, the present study represents a preliminary attempt to assess this potential as achieved by examining changes in the morphology and functionally related proteins of MAMs in a DE mouse model. We found that within the hippocampus of DE mice, there was a dyshomeostasis in the area of MAM. At present, some studies have also proposed that in the diabetic model, the homeostatic changes of MAMs cause calcium overload, affect mitochondrial function, and lead to cognitive impairment. For example, in C57BL/6J mice with an 8-week course of diabetes induced by STZ, there are hippocampus injuries and cognitive impairment, accompanied by mitochondrial Ca^2+^ overload-induced mitochondrial dysfunction and apoptosis [41]. And, Li et al. also found that calcium overload and cognitive impairment appeared in db/db mice [42]. Although they found that MAMs increase in the diabetic state, which is inconsistent with the reduction of MAMs in STZ-induced DE mice with a 12-week course of diabetes in our study, this may be due to differences in animal models and the duration of disease states. Importantly, MAMs changed in all studies compared to controls, with either increases or decreases, representing a manifestation of homeostatic imbalance. MAMs participate in lipid metabolism, autophagy, ER stress, calcium homeostasis, and so on, and the homeostasis of MAMs is important to maintain normal function [43]. Calcium overload occurs when MAMs formation is promoted, leading to cognitive impairment. However, when MAMs integrity is impaired, cholesterol turnover is inhibited, causing cognitive deficits too [44]. These results suggest that homeostatic imbalance in MAMs is a pathological condition in which a state of normal MAMs functioning is difficult to maintain.

In addition, we found that the expression of phosphatidylserine synthetase 1 (PSS1), an important protein involved in phospholipid metabolism within MAMs, was significantly increased in DE mice. PSS1 is responsible for the synthesis of phosphatidylserine (PS), which is an essential component of neuronal cell membranes required for the maintenance of normal cognitive functions [45, 46]. Interestingly, PS synthesis is co-regulated by both Lipin1 and PSS1 pathways. CHO1-encoded phosphatidylserine synthetase (PSS) and PAH1-encoded phosphatidic acid phosphatase (PAP) are involved in controlling the balance between membrane phospholipids and triglyceride synthesis through two distinct pathways. With a loss of PAP activity, PSS activity is increased to enhance phosphatidic acid levels [47]. In the present study, a decrease in Lipin1, which possesses PAP activity, was accompanied with a significant increase in PSS1 in DE mice. And, as reported previously by our group, findings from our lipidomic analysis revealed that neuronal cells treated with high concentrations of glucose were accompanied with a reduction of PS [32]. Related to these findings, is the belief that increases in PSS1 may represent a compensatory mechanism following a decrease in Lipin1 expression due to high levels of glucose. However, this compensatory response does not completely reverse the phospholipid disorder observed. Thus, disruption of Lipin1 and PSS1 may represent critical factor involved with the imbalance in MAMs homeostasis within the hippocampus of DE mice.

Results from previous studies have demonstrated that endoplasmic reticulum stress affects lipid metabolism and can produce an accumulation of lipids [48, 49], which in turn affects the structure and function of MAMs. Lipin exerts an influence on MAMs by affecting phospholipid metabolism in endoplasmic reticulum. For example, Lipin1 deficiency causes endoplasmic reticulum stress. Lipin1 deficiency causes endoplasmic reticulum stress in skeletal muscle [50] and in the myocardium of lipin-1 deficient (fld) mice, decreased PAP activity and an accumulation of phosphatidic acid, all of which are associated with increased endoplasmic reticulum stress [51]. Lipin1 silencing induces an activation of endoplasmic reticulum stress through the IRE1α pathway in breast and lung cancer cells [52, 53]. The results of our present study are in accord with these findings.

Mitochondria, as another organelle involved in the composition of MAMs, are important for the regulation of neural and cognitive functions [54, 55]. Mitophagy, the selective degradation of damaged or excessive numbers of mitochondria [56], is a safeguard for the maintenance of normal mitochondrial function. In our study, it was found that Lipin1 deficiency leads to an increase in mitophagy. The findings of Fan et al. showing that knockdown of Lipin1 in lung cancer cells induced an initiation of autophagy, support the results of our study [52]. However, in Lipin1-deficient myopathies, autophagic precursor formation is impaired, autophagic flux is blocked and mitophagy is dysregulated [19, 29, 57]. Mitophagy activates mitochondrial autophagosome formation via Lipin1-mediated generation of mitochondrial DAG in Hela cells [58]. These findings are somewhat in contrast to our present results. A possible reason may be due to differences in the conditions in which Lipin1 alteration and autophagy occur. In DE mice of this experiment, reduced Lipin1 was not present from birth but was an acquired change, which may have had a complex effect on the changes in mitophagy. Therefore, more in-depth studies will be required to elucidate the specific relationship between Lipin1 and mitophagy.

We also found that Lipin1 affects mitochondrial oxidative stress. Specifically, our results demonstrated that mitochondrial oxidative stress was enhanced in DE and after interference with Lipin1 expression, and reduced with an overexpression of Lipin1 in DE mice. Similarly, in patients with Lipin1 mutations, their Lipin1 deficiency can lead to severe rhabdomyolysis, symptoms associated with oxidative stress [59]. Oxidative stress is related to pathways such as MAPK, NF-κB, and p53 [60–62]. Lipin1 may regulate the production of ROS by influencing these pathways. Moreover, Lipin1 is closely related to AMPK. For instance, the SIRT1-AMPK axis participates in the regulation of lipid metabolism through its interaction with Lipin1 [63]. AMPK also regulates NFκB, which is involved in the regulation of oxidative stress, autophagy and other processes [61]. And Lipin1 also regulates NFκB, thereby influencing cellular functions [26, 64]. In studies directed at investigating the regulation of fatty acid oxidation by p53 and Lipin1 in tumor cells, Lipin1 expression was induced in a p53-dependent manner. Moreover, an activation of intracellular ROS was observed during glucose deprivation, and Lpin1 expression is controlled through the ROS-ATM-p53 pathway [65]. In the offspring of mothers fed a high-fat diet during the prenatal period, hepatic oxidative stress was induced in these offspring leading to hypermethylation of the TF-binding site upstream of Lipin1, an effect which coincided with a decrease in Lipin1 mRNA expression [66]. All of these studies have reflected the close relationship between Lipin1 and oxidative stress. Lipin1 can be ultimately involved in the regulation of oxidative stress by participating in relevant pathways.

Neuroinflammation plays an important role in neurodegenerative diseases. As a part of this study, which was focused upon examining the pathogenesis of DE, we also investigated the relationship between Lipin1 and inflammation. Our findings revealed that Lipin1 was negatively correlated with inflammation in the nervous system. Similarly, in muscle tissue, a Lipin1 deficiency was associated with increased inflammation, a finding which is consistent with our present findings. In Duchenne muscular dystrophy there is a reduction in Lipin1. Jama et al. reported that inflammatory markers were increased in mdx mice, muscle-specific Lipin1-deficient mice and, in particular, substantially increased in dystrophin/lipin1double knockout (DKO) mice [67]. Recent evidence has shown that lipin1 has potential anti-inflammatory properties. A Lipin1 deficiency in myoblasts leads to an accumulation of oxidized mitochondrial DNA in endosomes, an effect which then activates the toll-like receptor 9 (TLR9) along with triggering inflammatory signals and a caspase-dependent rhabdomyolysis [29]. Lipin1 inhibits the expression of pro-inflammatory cytokines in adipocytes by suppressing the activity of NFATc4 [64]. In the liver, ablation of Lipin1 activates NFATc4 and NF-κB, thereby significantly increasing the expression of pro-inflammatory factors [26]. In hepatocytes, lipin-1α is mainly located in the nucleus, whereas lipin-1β is mainly located in the cytoplasm. Deletion of SIRT1 in mouse hepatocytes disrupts Lipin1 signaling and increases the Lipin1β/α ratio, resulting in inflammatory responses and exacerbating alcoholic fatty liver [68]. This suggests that alterations in the intracellular distribution of Lipin1 can have diverse effects on the cell. However, in studies on skin cells, UVB radiation resulted in a decreased expression of Lipin1 in NHEK cells. With the downregulation of Lipin1, the accumulation of free fatty acids induced by UVB is inhibited, and NF-κB phosphorylation is ameliorated. Moreover, this inhibition of Lipin1 is protective against UVB-induced pro-inflammatory responses [69]. Taken together, it appears that the exact relationship between Lipin1 and inflammation is critically contingent upon the tissue affected. In different types of cells, the impact of Lipin1 alteration on lipid metabolism varies, potentially influencing pathways differently and ultimately leading to different pathological directions.

A notable limitation of this study that will require further investigation is that the specific upstream and downstream mechanisms among the various relationships reported here (that the link of MAMs disorder to endoplasmic reticulum stress and mitochondrial dysfunction) remain to be verified and elucidated.

## Conclusions

A Lipin1 deficiency, as resulting from chronic hyperglycemia, contributes to an imbalance of MAMs homeostasis in hippocampal neurons. These effects are accompanied with an enhancement of ER stress and mitochondrial dysfunction, eventually leading to the cognitive deficits observed in DE. In addition, low levels of hippocampal Lipin1 activate microglia and increase inflammation in a DE mouse model. These changes, as observed in this DE model, were ameliorated after increasing Lipin1 expression.

## Electronic supplementary material

Below is the link to the electronic supplementary material.

Supplementary Material 1: Fig. S1. Water intake, food consumption and urine output in DE mice. (A) Water intake of mice before and after injection (*n* = 5 per group). (B) Food consumption of mice before and after injection (*n* = 5 per group). (C) Urine output of mice before and after injection (*n* = 5 per group). All data are shown as means ± SEMs. ****p < 0.0001, Ctrl vs. DE. Ctrl, Control. DE, Diabetic Encephalopathy.

Supplementary Material 2: Table S1-S7. One-way ANCOVA of escape latencies in control and DE mice.

Supplementary Material 3: Fig. S2. Lipin1 expression within hippocampal neurons, astrocytes and microglia in DE mice. (A) Fluorescent images showing co-localizations of Lipin1 and NeuN. Scale bar is 10 μm and 5 μm. (B) Lipin1^+^/NeuN^+^ colocalization in fluorescent images (*n* = 9 per group). (C) Fluorescent images showing co-localizations of Lipin1 and GFAP. Scale bar is 5 μm. (D) Lipin1^+^/GFAP^+^ colocalization in fluorescent images (*n* = 9 cells per group). (E) Fluorescent images showing co-localizations of Lipin1 and Iba1. Scale bar is 5 μm. (F) Lipin1^+^/Iba1^+^ colocalization in fluorescent images (*n* = 9 cells per group). All data are shown as means ± SEMs. **p < 0.01, Ctrl vs. DE. Ctrl, Control. DE, Diabetic Encephalopathy.

Supplementary Material 4: Fig. S3. The mitochondrial function was impaired in neurons treated with high glucose. (A) Representative immunofluorescent images of TMRM. Scale bar is 50 μm. (B) Quantitative analysis of the relative fluorescence intensity of TMEM (*n* = 3 per group). (C) Representative immunofluorescent images of Mito-SOX. Scale bar is 50 μm. (D) Quantitative analysis of the relative fluorescence intensity of Mito-SOX (*n* = 3 per group). (E) Representative immunofluorescent images of mitochondrial length. Scale bar is 10 μm. (F) Analysis of the relative length of mitochondria. All data are shown as means ± SEMs. *p < 0.05, **p < 0.01, and ***p < 0.001, 25G vs. 100G.

Supplementary Material 5: Fig. S4. Regulation of Lipin1 Expression by AAV in Animal Model. (A) RT-PCR assays of mRNA expression levels of Lipin1 after AAV injection (*n* = 9 per group). (B) Representative Western blot images showing relative protein expression of Lipin1 after AAV injection (*n* = 6 per group).

Supplementary Material 6: Table S8-S12. One-way ANCOVA of escape latencies in models that regulate Lipin1.

Supplementary Material 7: Fig.S5. The level of oxidative stress after mitochondrial mass control. (A) Representative immunofluorescent images of Mito-SOX and Tom20. Scale bar is 8 μm. (B) Relative levels of oxidative stress of mitochondrial mass control (*n* = 9 per group). All data are shown as means ± SEMs. ****p < 0.0001, WT + AAV-Ctrl vs. Other groups. ^####^*p* < 0.0001, DE + AAV-Lipin1 vs. Other groups.

Supplementary Material 8: Fig. S6. Regulation of Lipin1 Expression by LV in neuron. (A) RT-PCR assays of mRNA expression levels of Lipin1 after LV injection in HT22 cells (*n* = 10 per group). (B) Representative Western blot images showing relative protein expressions after LV injection in HT22 cells for Lipin1 (*n* = 8 per group).

Supplementary Material 9

Supplementary Material 10

## Data Availability

Data is provided within the supplementary information files.

## Abbreviations

AAV: Adeno-associated virus
CHOP: C/EBP-homologous protein
DAG: Diacylglycerol
DE: Diabetic encephalopathy
MAMs: Mitochondria-associated endoplasmic reticulum membranes
MG: Methylglyoxal
MWM: Morris water maze
ER: Endoplasmic reticulum
GRP78: 78-kD glucose-regulated protein
PA: Phosphatidate
PAP: Phosphatidate phosphatase
PFA: Paraformaldehyde
PINK: PTEN Induced putative Kinase 1
PKC: Protein kinase C
PKD: Protein kinase D
RBG: Random blood glucose
PE: phosphatidylethanolamine
PS: Phosphatidylserine
PSS: Phosphatidylserine synthetase 1
RT-PCR: real‑time quantitative PCR
TEM: Transmission electron microscope
TLR9: Toll-like receptor 9
T1DM: Type 1 diabetes mellitus
T2DM: Type 2 diabetes mellitus
