# Supplementary figures and images for: Phosphatidate phosphatase Lipin1 alters mitochondria-associated endoplasmic reticulum membranes (MAMs) homeostasis: effects which contribute to the development of diabetic encephalopathy
Source: J Neuroinflammation. 2025 Apr 18;22:111. doi: 10.1186/s12974-025-03441-3 (PMC12008933; doi:10.1186/s12974-025-03441-3)

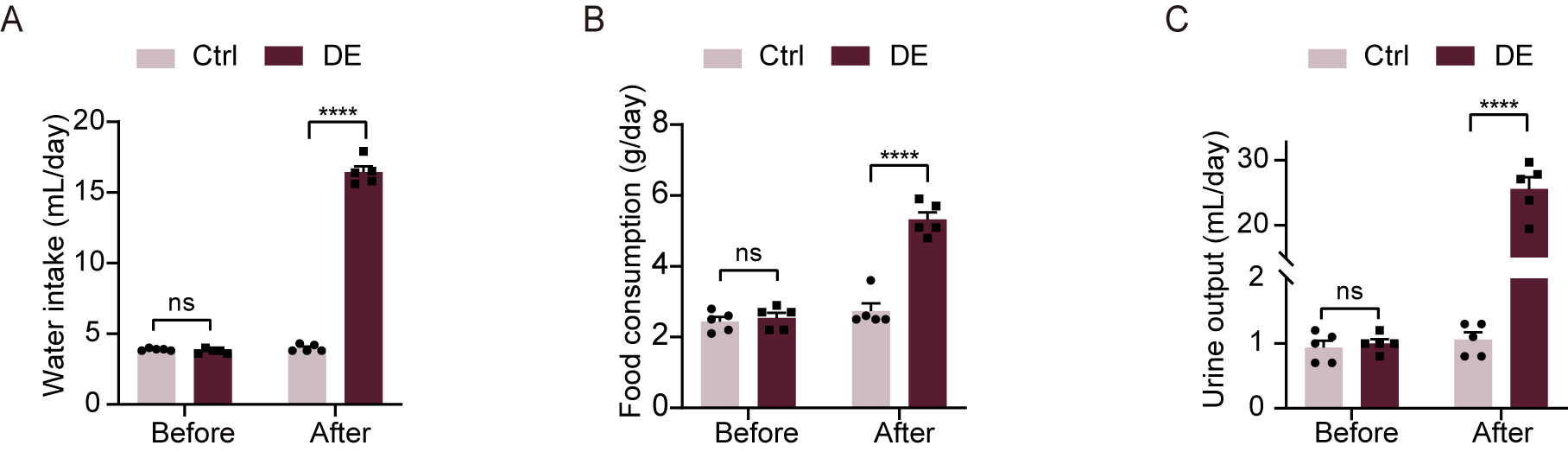

Supplement: Supplementary file 1 — Supplementary Material 1: Fig. S1. Water intake, food consumption and urine output in DE mice. (A) Water intake of mice before and after injection (n = 5 per group). (B) Food consumption of mice before and after injection (n = 5 per group). (C) Urine output of mice before and after injection (n = 5 per group). All data are shown as means ± SEMs. ****p < 0.0001, Ctrl vs. DE. Ctrl, Control. DE, Diabetic Encephalopathy. [file 12974_2025_3441_MOESM1_ESM.tif]

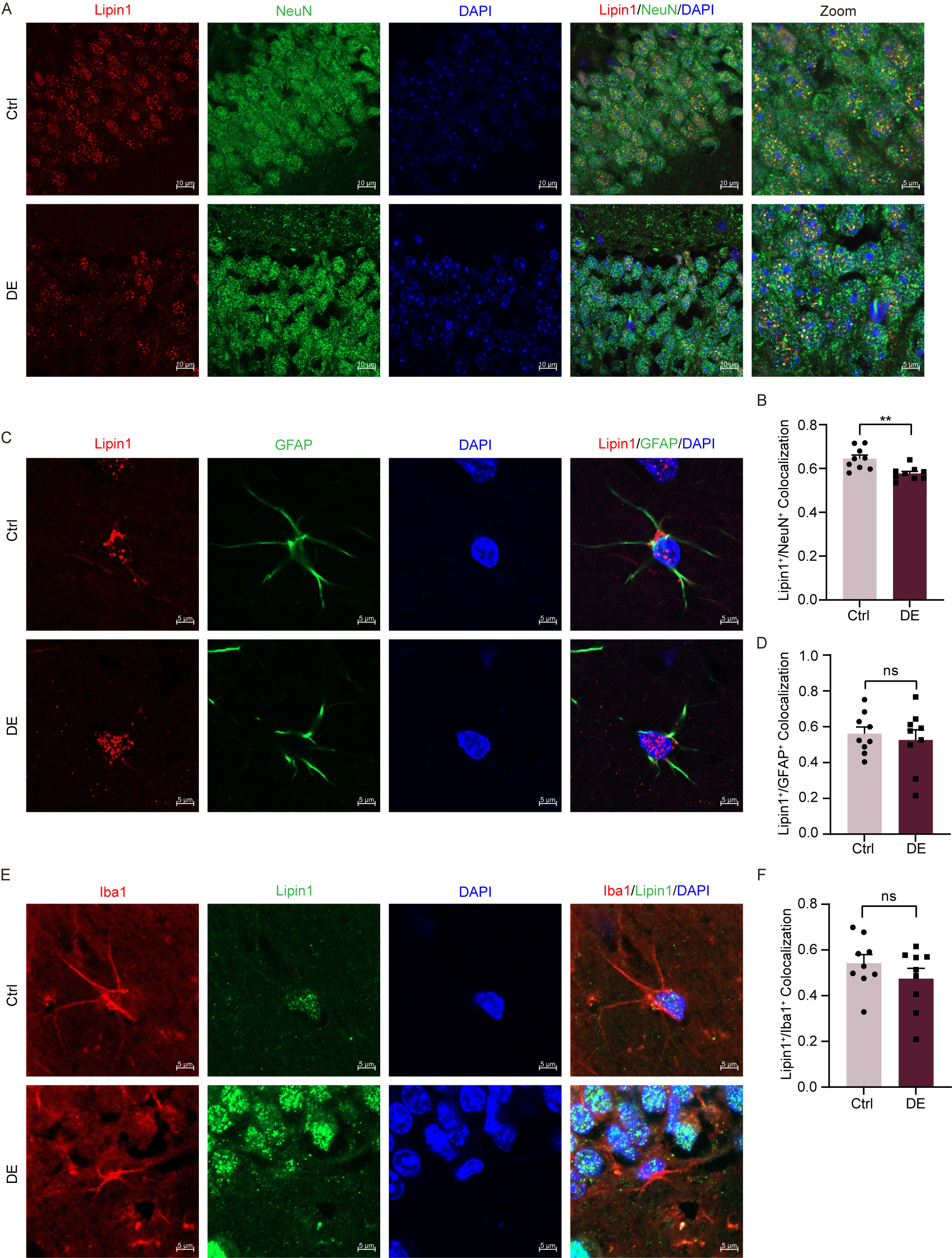

Supplement: Supplementary file 3 — Supplementary Material 3: Fig. S2. Lipin1 expression within hippocampal neurons, astrocytes and microglia in DE mice. (A) Fluorescent images showing co-localizations of Lipin1 and NeuN. Scale bar is 10 μm and 5 μm. (B) Lipin1+/NeuN+ colocalization in fluorescent images (n = 9 per group). (C) Fluorescent images showing co-localizations of Lipin1 and GFAP. Scale bar is 5 μm. (D) Lipin1+/GFAP+ colocalization in fluorescent images (n = 9 cells per group). (E) Fluorescent images showing co-localizations of Lipin1 and Iba1. Scale bar is 5 μm. (F) Lipin1+/Iba1+ colocalization in fluorescent images (n = 9 cells per group). All data are shown as means ± SEMs. **p < 0.01, Ctrl vs. DE. Ctrl, Control. DE, Diabetic Encephalopathy. [file 12974_2025_3441_MOESM3_ESM.tif]

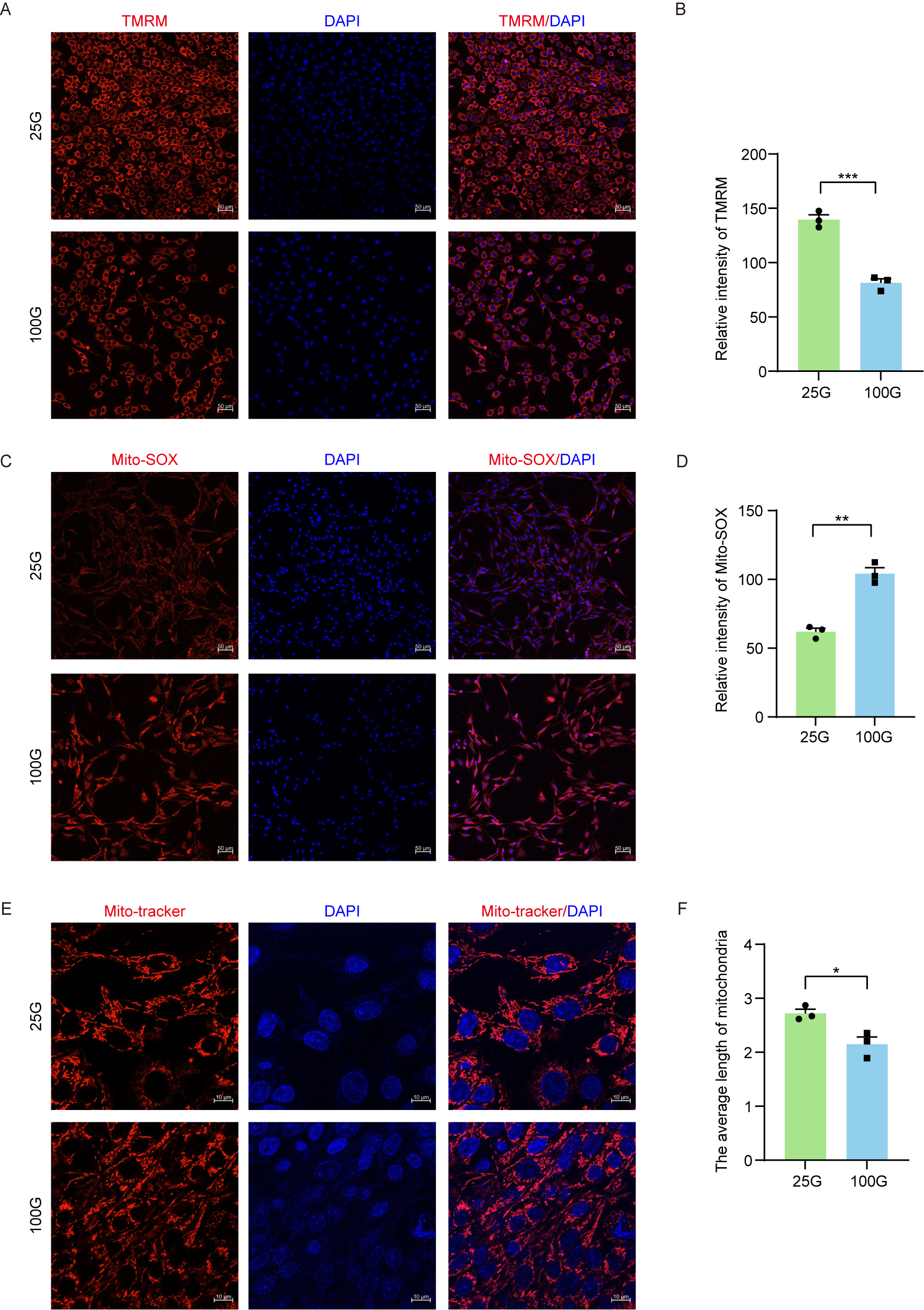

Supplement: Supplementary file 4 — Supplementary Material 4: Fig. S3. The mitochondrial function was impaired in neurons treated with high glucose. (A) Representative immunofluorescent images of TMRM. Scale bar is 50 μm. (B) Quantitative analysis of the relative fluorescence intensity of TMEM (n = 3 per group). (C) Representative immunofluorescent images of Mito-SOX. Scale bar is 50 μm. (D) Quantitative analysis of the relative fluorescence intensity of Mito-SOX (n = 3 per group). (E) Representative immunofluorescent images of mitochondrial length. Scale bar is 10 μm. (F) Analysis of the relative length of mitochondria. All data are shown as means ± SEMs. *p < 0.05, **p < 0.01, and ***p < 0.001, 25G vs. 100G. [file 12974_2025_3441_MOESM4_ESM.tif]

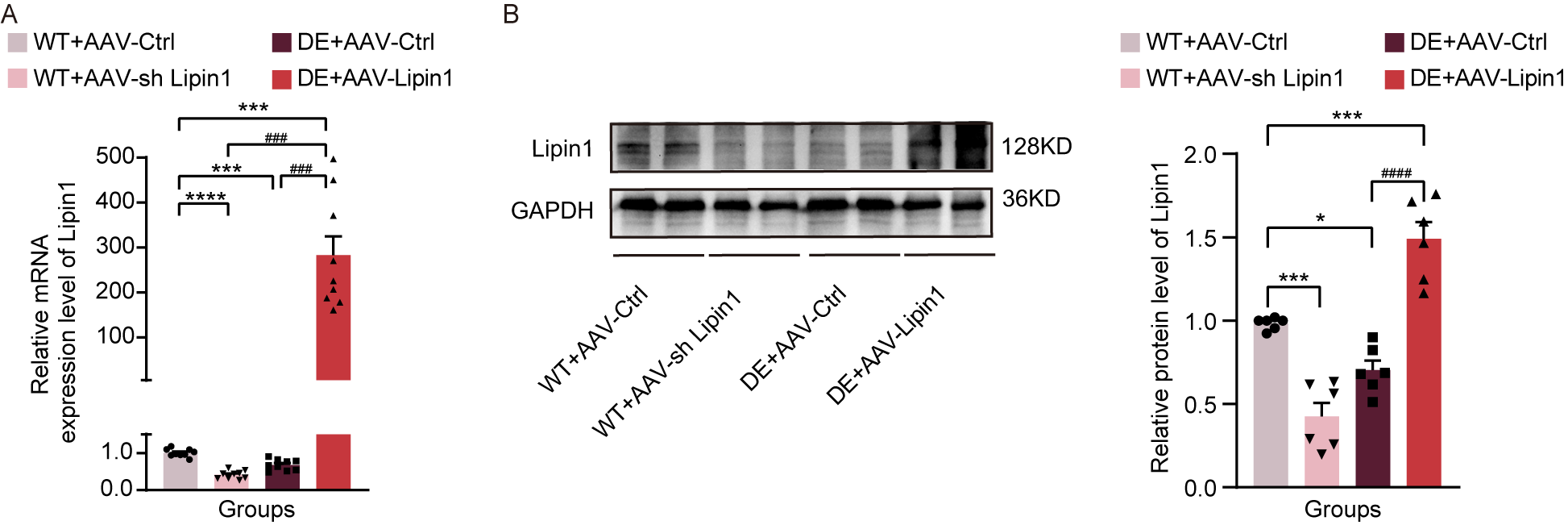

Supplement: Supplementary file 5 — Supplementary Material 5: Fig. S4. Regulation of Lipin1 Expression by AAV in Animal Model. (A) RT-PCR assays of mRNA expression levels of Lipin1 after AAV injection (n = 9 per group). (B) Representative Western blot images showing relative protein expression of Lipin1 after AAV injection (n = 6 per group). [file 12974_2025_3441_MOESM5_ESM.tif]

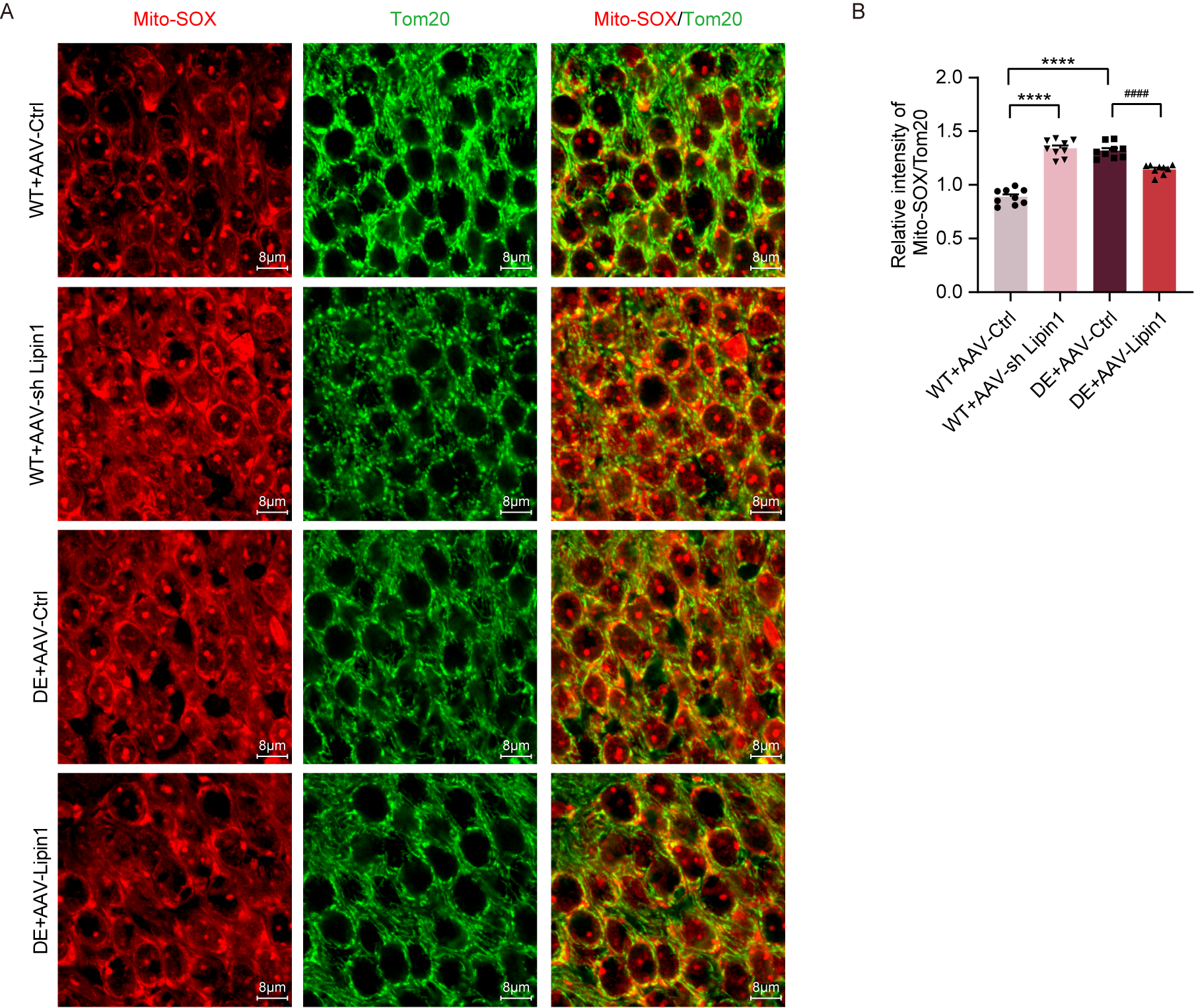

Supplement: Supplementary file 7 — Supplementary Material 7: Fig.S5. The level of oxidative stress after mitochondrial mass control. (A) Representative immunofluorescent images of Mito-SOX and Tom20. Scale bar is 8 μm. (B) Relative levels of oxidative stress of mitochondrial mass control (n = 9 per group). All data are shown as means ± SEMs. ****p < 0.0001, WT + AAV-Ctrl vs. Other groups. ####p < 0.0001, DE + AAV-Lipin1 vs. Other groups. [file 12974_2025_3441_MOESM7_ESM.tif]

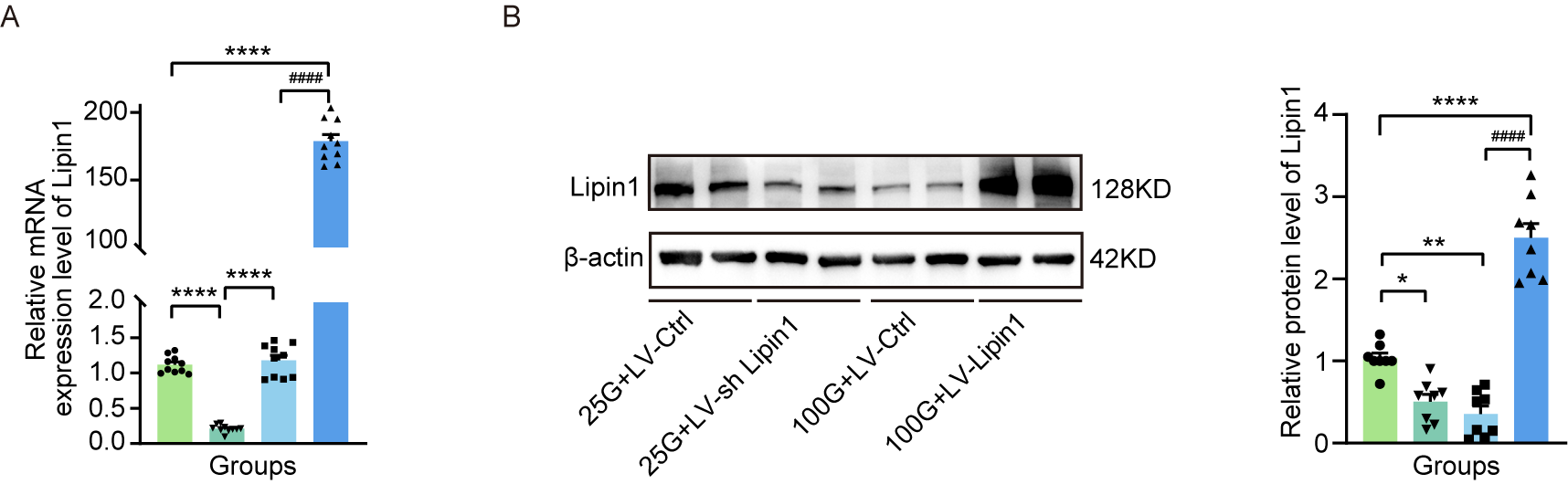

Supplement: Supplementary file 8 — Supplementary Material 8: Fig. S6. Regulation of Lipin1 Expression by LV in neuron. (A) RT-PCR assays of mRNA expression levels of Lipin1 after LV injection in HT22 cells (n = 10 per group). (B) Representative Western blot images showing relative protein expressions after LV injection in HT22 cells for Lipin1 (n = 8 per group). [file 12974_2025_3441_MOESM8_ESM.tif]
