# Supplementary material for: Phosphatidate phosphatase Lipin1 alters mitochondria-associated endoplasmic reticulum membranes (MAMs) homeostasis: effects which contribute to the development of diabetic encephalopathy
Source: J Neuroinflammation. 2025 Apr 18;22:111. doi: 10.1186/s12974-025-03441-3 (PMC12008933; doi:10.1186/s12974-025-03441-3)
Supplement: Supplementary file 2 — Supplementary Material 2: Table S1-S7. One-way ANCOVA of escape latencies in control and DE mice. [file 12974_2025_3441_MOESM2_ESM.docx]

Table S1. One-way ANCOVA of escape latencies in control and DE mice (Day 1)

| **Tests of Between-Subjects Effects**  Dependent Variable: Escape Latencies (Day 1) | | | | | | |
| --- | --- | --- | --- | --- | --- | --- |
| Source | Type III Sum of Squares | df | Mean Square | F | Sig. | Partial Eta Squared |
| Corrected Model | 275.993^a^ | 2 | 137.997 | 1.818 | 0.182 | 0.119 |
| Intercept | 2189.539 | 1 | 2189.539 | 28.850 | 0.000 | 0.517 |
| Groups | 177.482 | 1 | 177.482 | 2.339 | 0.138 | 0.080 |
| Speed | 21.368 | 1 | 21.368 | 0.282 | 0.600 | 0.010 |
| Error | 2049.115 | 27 | 75.893 |  |  |  |
| Total | 85707.060 | 30 |  |  |  |  |
| Corrected Total | 2325.108 | 29 |  |  |  |  |

a. R Squared = 0.119 (Adjusted R Squared = 0.053)

Table S2. One-way ANCOVA of escape latencies in control and DE mice (Day 2)

| **Tests of Between-Subjects Effects**  Dependent Variable: Escape Latencies (Day 2) | | | | | | |
| --- | --- | --- | --- | --- | --- | --- |
| Source | Type III Sum of Squares | df | Mean Square | F | Sig. | Partial Eta Squared |
| Corrected Model | 4867.986^a^ | 2 | 2433.993 | 30.215 | 0.000 | 0.691 |
| Intercept | 2145.761 | 1 | 2145.761 | 26.637 | 0.000 | 0.497 |
| Groups | 1607.888 | 1 | 1607.888 | 19.960 | 0.000 | 0.425 |
| Speed | 6.399 | 1 | 6.399 | 0.079 | 0.780 | 0.003 |
| Error | 2175.001 | 27 | 80.556 |  |  |  |
| Total | 67819.990 | 30 |  |  |  |  |
| Corrected Total | 7042.987 | 29 |  |  |  |  |

a. R Squared = 0.691 (Adjusted R Squared = 0.668)

Table S3. One-way ANCOVA of escape latencies in control and DE mice (Day 3)

| **Tests of Between-Subjects Effects**  Dependent Variable: Escape Latencies (Day 3) | | | | | | |
| --- | --- | --- | --- | --- | --- | --- |
| Source | Type III Sum of Squares | df | Mean Square | F | Sig. | Partial Eta Squared |
| Corrected Model | 6189.665^a^ | 2 | 3094.832 | 30.104 | 0.000 | 0.690 |
| Intercept | 1967.939 | 1 | 1967.939 | 19.142 | 0.000 | 0.415 |
| Groups | 1920.536 | 1 | 1920.536 | 18.681 | 0.000 | 0.409 |
| Speed | 20.597 | 1 | 20.597 | 0.200 | 0.658 | 0.007 |
| Error | 2775.742 | 27 | 102.805 |  |  |  |
| Total | 59313.440 | 30 |  |  |  |  |
| Corrected Total | 8965.407 | 29 |  |  |  |  |

a. R Squared = 0.690 (Adjusted R Squared = 0.667)

Table S4. One-way ANCOVA of escape latencies in control and DE mice (Day 4)

| **Tests of Between-Subjects Effects**  Dependent Variable: Escape Latencies (Day 4) | | | | | | |
| --- | --- | --- | --- | --- | --- | --- |
| Source | Type III Sum of Squares | df | Mean Square | F | Sig. | Partial Eta Squared |
| Corrected Model | 7821.612^a^ | 2 | 3910.806 | 111.142 | 0.000 | 0.892 |
| Intercept | 2809.022 | 1 | 2809.022 | 79.830 | 0.000 | 0.747 |
| Groups | 1455.059 | 1 | 1455.059 | 41.352 | 0.000 | 0.605 |
| Speed | 316.603 | 1 | 316.603 | 8.998 | 0.006 | 0.250 |
| Error | 950.062 | 27 | 35.187 |  |  |  |
| Total | 48615.170 | 30 |  |  |  |  |
| Corrected Total | 8771.674 | 29 |  |  |  |  |

a. R Squared = 0.892 (Adjusted R Squared = 0.884)

Table S5. One-way ANCOVA of escape latencies in control and DE mice (Day 5)

| **Tests of Between-Subjects Effects**  Dependent Variable: Escape Latencies (Day 5) | | | | | | |
| --- | --- | --- | --- | --- | --- | --- |
| Source | Type III Sum of Squares | df | Mean Square | F | Sig. | Partial Eta Squared |
| Corrected Model | 7792.318^a^ | 2 | 3896.159 | 36.227 | 0.000 | 0.729 |
| Intercept | 2274.184 | 1 | 2274.184 | 21.146 | 0.000 | 0.439 |
| Groups | 1547.438 | 1 | 1547.438 | 14.388 | 0.001 | 0.348 |
| Speed | 268.318 | 1 | 268.318 | 2.495 | 0.126 | 0.085 |
| Error | 2903.824 | 27 | 107.549 |  |  |  |
| Total | 42221.350 | 30 |  |  |  |  |
| Corrected Total | 10696.142 | 29 |  |  |  |  |

a. R Squared = 0.729 (Adjusted R Squared = 0.708)

Table S6. Estimated Marginal Means-Estimates in control and DE mice (Day 4)

| **Estimates**  Dependent Variable: Escape Latencies (Day 4) | | | | |
| --- | --- | --- | --- | --- |
| Groups | Means | Std. Error | 95% Confidence Interval | |
|  |  |  | Lower Bound | Upper Bound |
| Ctrl | 24.913^a^ | 2.095 | 20.615 | 29.211 |
| DE | 47.974^a^ | 2.095 | 43.675 | 52.272 |

a. Covariates appearing in the model are evaluated at the following values: Speed = 0.9305.

Table S7. Estimated Marginal Means-Pairwise Comparisons in control and DE mice (Day 4)

| **Pairwise Comparisons**  Dependent Variable: Escape Latencies (Day 4) | | | | | | |
| --- | --- | --- | --- | --- | --- | --- |
| Groups | Groups | Mean Difference | Std. Error | Sig. | 95% Confidence Interval for Difference | |
|  |  |  |  |  | Lower Bound | Upper Bound |
| Ctrl | DE | -23.060 | 3.586 | 0.000 | -30.418 | -15.702 |

Based on estimated marginal means.
