# Supplementary material for: Phosphatidate phosphatase Lipin1 alters mitochondria-associated endoplasmic reticulum membranes (MAMs) homeostasis: effects which contribute to the development of diabetic encephalopathy
Source: J Neuroinflammation. 2025 Apr 18;22:111. doi: 10.1186/s12974-025-03441-3 (PMC12008933; doi:10.1186/s12974-025-03441-3)
Supplement: Supplementary file 6 — Supplementary Material 6: Table S8-S12. One-way ANCOVA of escape latencies in models that regulate Lipin1. [file 12974_2025_3441_MOESM6_ESM.docx]

Table S8. One-way ANCOVA of escape latencies in models that regulate Lipin1 (Day 1)

| **Tests of Between-Subjects Effects**  Dependent Variable: Escape Latencies (Day 1) | | | | | | |
| --- | --- | --- | --- | --- | --- | --- |
| Source | Type III Sum of Squares | df | Mean Square | F | Sig. | Partial Eta Squared |
| Corrected Model | 791.844^a^ | 4 | 197.961 | 1.661 | 0.183 | 0.168 |
| Intercept | 1958.750 | 1 | 1958.750 | 16.435 | 0.000 | 0.332 |
| Groups | 504.084 | 3 | 168.028 | 1.410 | 0.257 | 0.114 |
| Speed | 2.356 | 1 | 2.356 | 0.020 | 0.889 | 0.001 |
| Error | 3932.907 | 33 | 119.179 |  |  |  |
| Total | 88094.330 | 38 |  |  |  |  |
| Corrected Total | 4724.751 | 37 |  |  |  |  |

a. R Squared = 0.168 (Adjusted R Squared = 0.067)

Table S9. One-way ANCOVA of escape latencies in models that regulate Lipin1 (Day 2)

| **Tests of Between-Subjects Effects**  Dependent Variable: Escape Latencies (Day 2) | | | | | | |
| --- | --- | --- | --- | --- | --- | --- |
| Source | Type III Sum of Squares | df | Mean Square | F | Sig. | Partial Eta Squared |
| Corrected Model | 7439.472^a^ | 4 | 1859.868 | 21.650 | 0.000 | 0.724 |
| Intercept | 3027.272 | 1 | 3027.272 | 35.240 | 0.000 | 0.516 |
| Groups | 2688.582 | 3 | 896.194 | 10.432 | 0.000 | 0.487 |
| Speed | 246.456 | 1 | 246.456 | 2.869 | 0.100 | 0.080 |
| Error | 2834.850 | 33 | 85.905 |  |  |  |
| Total | 72701.060 | 38 |  |  |  |  |
| Corrected Total | 10274.322 | 37 |  |  |  |  |

a. R Squared = 0.724 (Adjusted R Squared = 0.691)

Table S10. One-way ANCOVA of escape latencies in models that regulate Lipin1 (Day 3)

| **Tests of Between-Subjects Effects**  Dependent Variable: Escape Latencies (Day 3) | | | | | | |
| --- | --- | --- | --- | --- | --- | --- |
| Source | Type III Sum of Squares | df | Mean Square | F | Sig. | Partial Eta Squared |
| Corrected Model | 8169.740^a^ | 4 | 2042.435 | 33.251 | 0.000 | 0.801 |
| Intercept | 1889.233 | 1 | 1889.233 | 30.757 | 0.000 | 0.482 |
| Groups | 3431.348 | 3 | 1143.783 | 18.621 | 0.000 | 0.629 |
| Speed | 78.144 | 1 | 78.144 | 1.272 | 0.267 | 0.037 |
| Error | 2026.989 | 33 | 61.424 |  |  |  |
| Total | 58563.110 | 38 |  |  |  |  |
| Corrected Total | 10196.729 | 37 |  |  |  |  |

a. R Squared = 0.801 (Adjusted R Squared = 0.777)

Table S11. One-way ANCOVA of escape latencies in models that regulate Lipin1 (Day 4)

| **Tests of Between-Subjects Effects**  Dependent Variable: Escape Latencies (Day 4) | | | | | | |
| --- | --- | --- | --- | --- | --- | --- |
| Source | Type III Sum of Squares | df | Mean Square | F | Sig. | Partial Eta Squared |
| Corrected Model | 13828.395^a^ | 4 | 3457.099 | 98.300 | 0.000 | 0.923 |
| Intercept | 1312.802 | 1 | 1312.802 | 37.328 | 0.000 | 0.531 |
| Groups | 7497.665 | 3 | 2499.222 | 71.063 | 0.000 | 0.866 |
| Speed | 9.714 | 1 | 9.714 | 0.276 | 0.603 | 0.008 |
| Error | 1160.573 | 33 | 35.169 |  |  |  |
| Total | 59114.390 | 38 |  |  |  |  |
| Corrected Total | 14988.969 | 37 |  |  |  |  |

a. R Squared = 0.923 (Adjusted R Squared = 0.913)

Table S12. One-way ANCOVA of escape latencies in models that regulate Lipin1 (Day 5)

| **Tests of Between-Subjects Effects**  Dependent Variable: Escape Latencies (Day 5) | | | | | | |
| --- | --- | --- | --- | --- | --- | --- |
| Source | Type III Sum of Squares | df | Mean Square | F | Sig. | Partial Eta Squared |
| Corrected Model | 14671.347^a^ | 4 | 3667.837 | 55.496 | 0.000 | 0.871 |
| Intercept | 883.546 | 1 | 883.546 | 13.369 | 0.001 | 0.288 |
| Groups | 7777.841 | 3 | 2592.614 | 39.228 | 0.000 | 0.781 |
| Speed | 5.677 | 1 | 5.677 | 0.086 | 0.771 | 0.003 |
| Error | 2181.024 | 33 | 66.092 |  |  |  |
| Total | 57832.750 | 38 |  |  |  |  |
| Corrected Total | 16852.371 | 37 |  |  |  |  |

a. R Squared = 0.871 (Adjusted R Squared = 0.855)
