# Supplementary material for: Phosphatidate phosphatase Lipin1 alters mitochondria-associated endoplasmic reticulum membranes (MAMs) homeostasis: effects which contribute to the development of diabetic encephalopathy
Source: J Neuroinflammation. 2025 Apr 18;22:111. doi: 10.1186/s12974-025-03441-3 (PMC12008933; doi:10.1186/s12974-025-03441-3)

Fig2.A

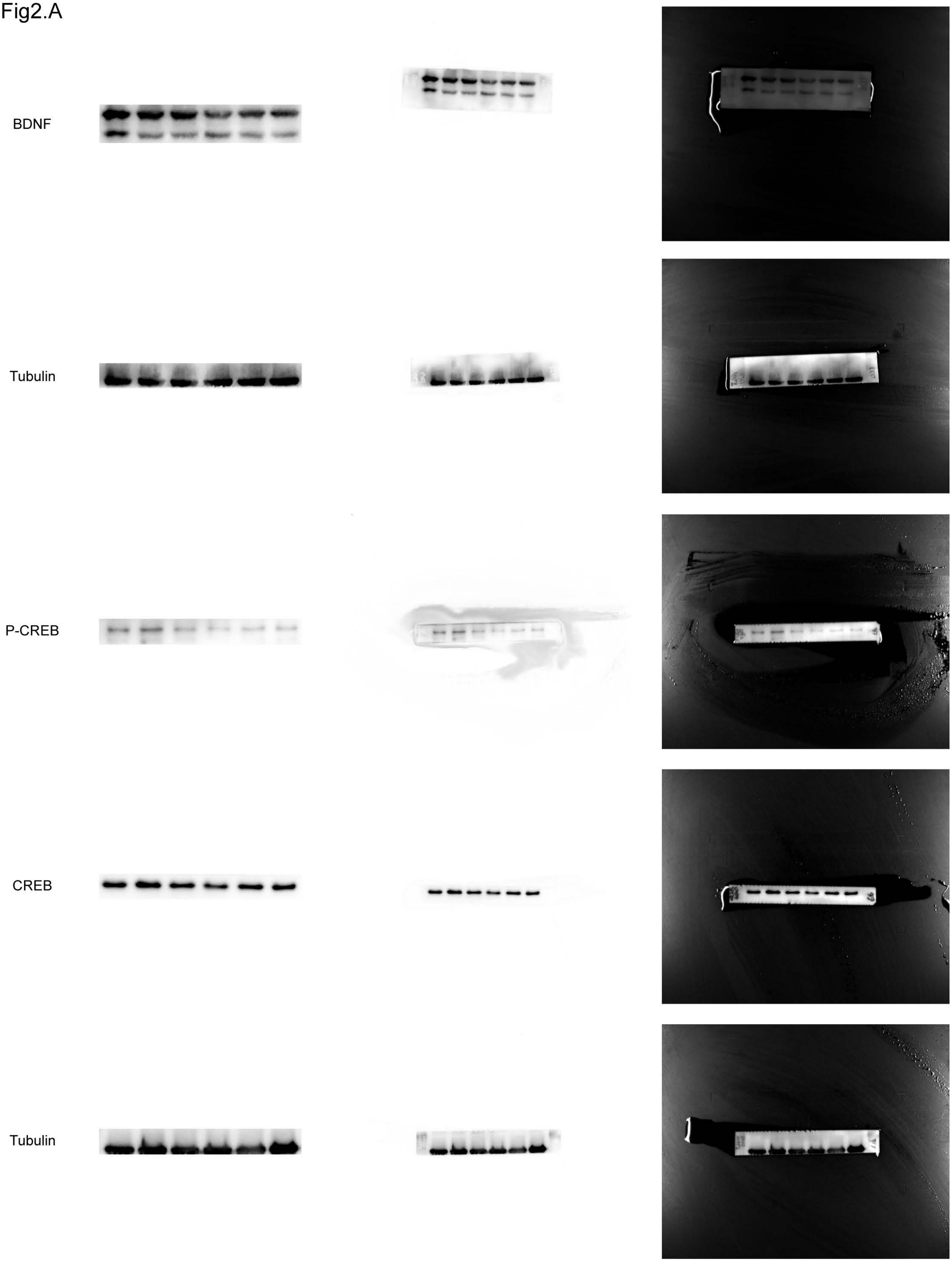

Fig2.D

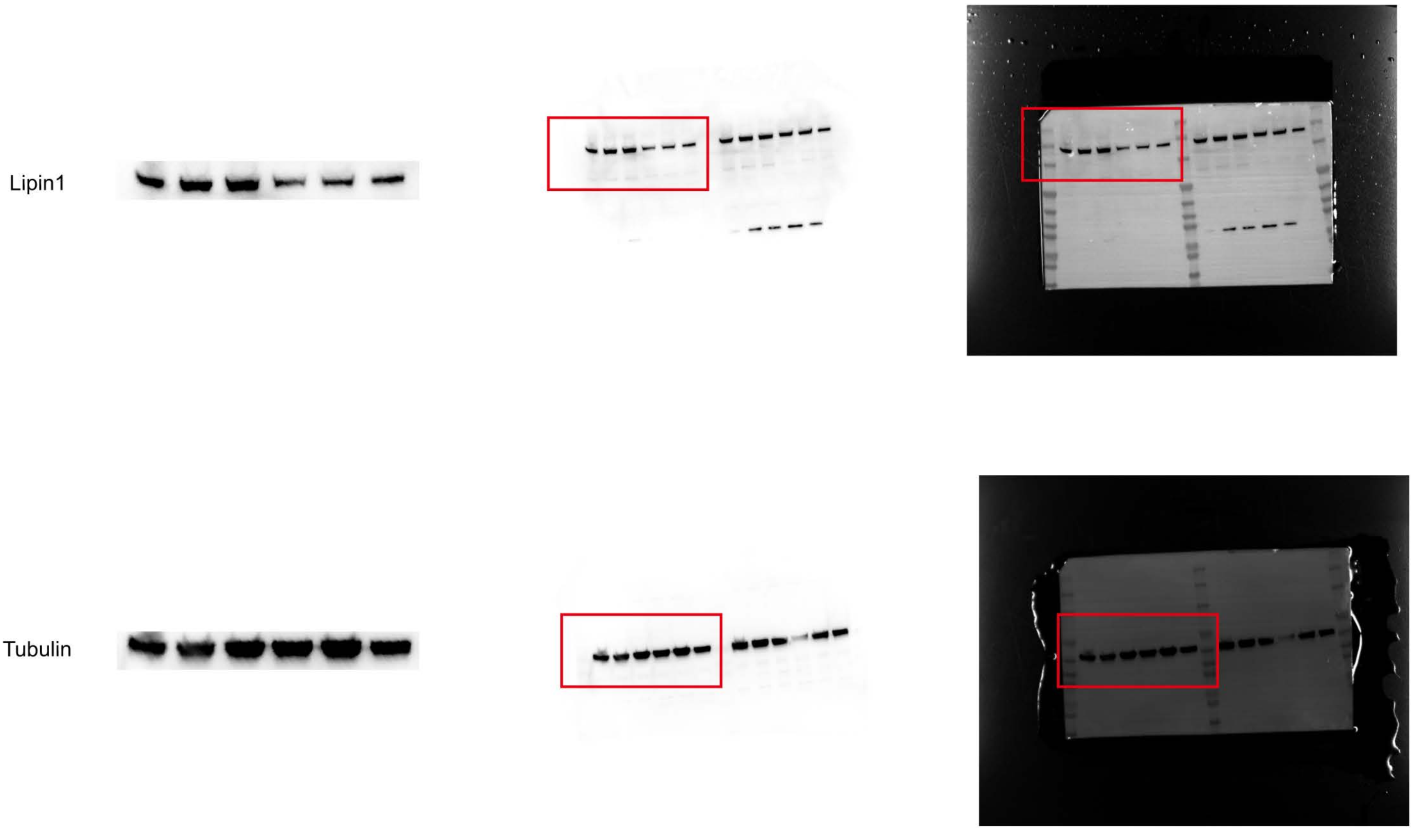

Fig3.D

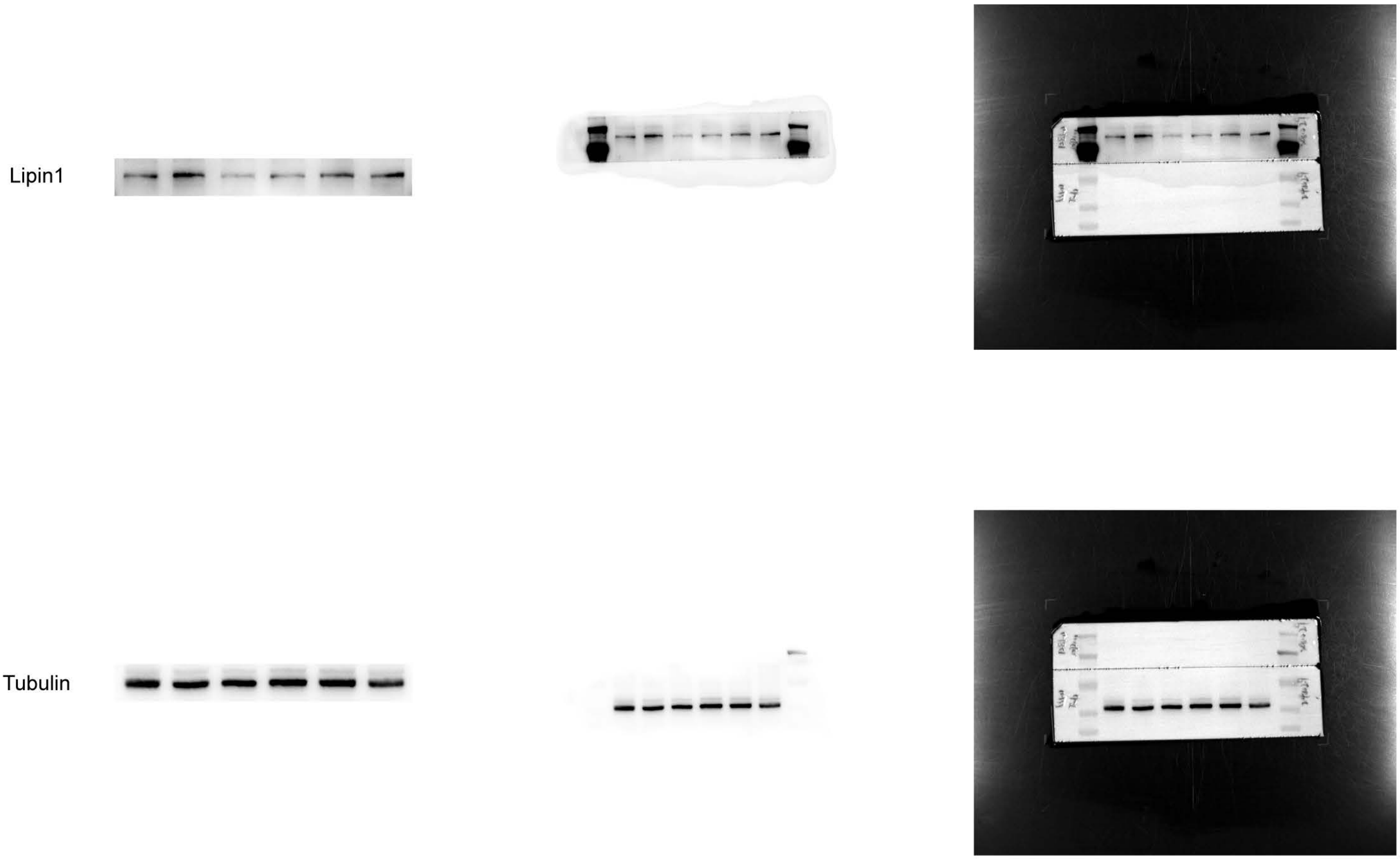

Fig5.A

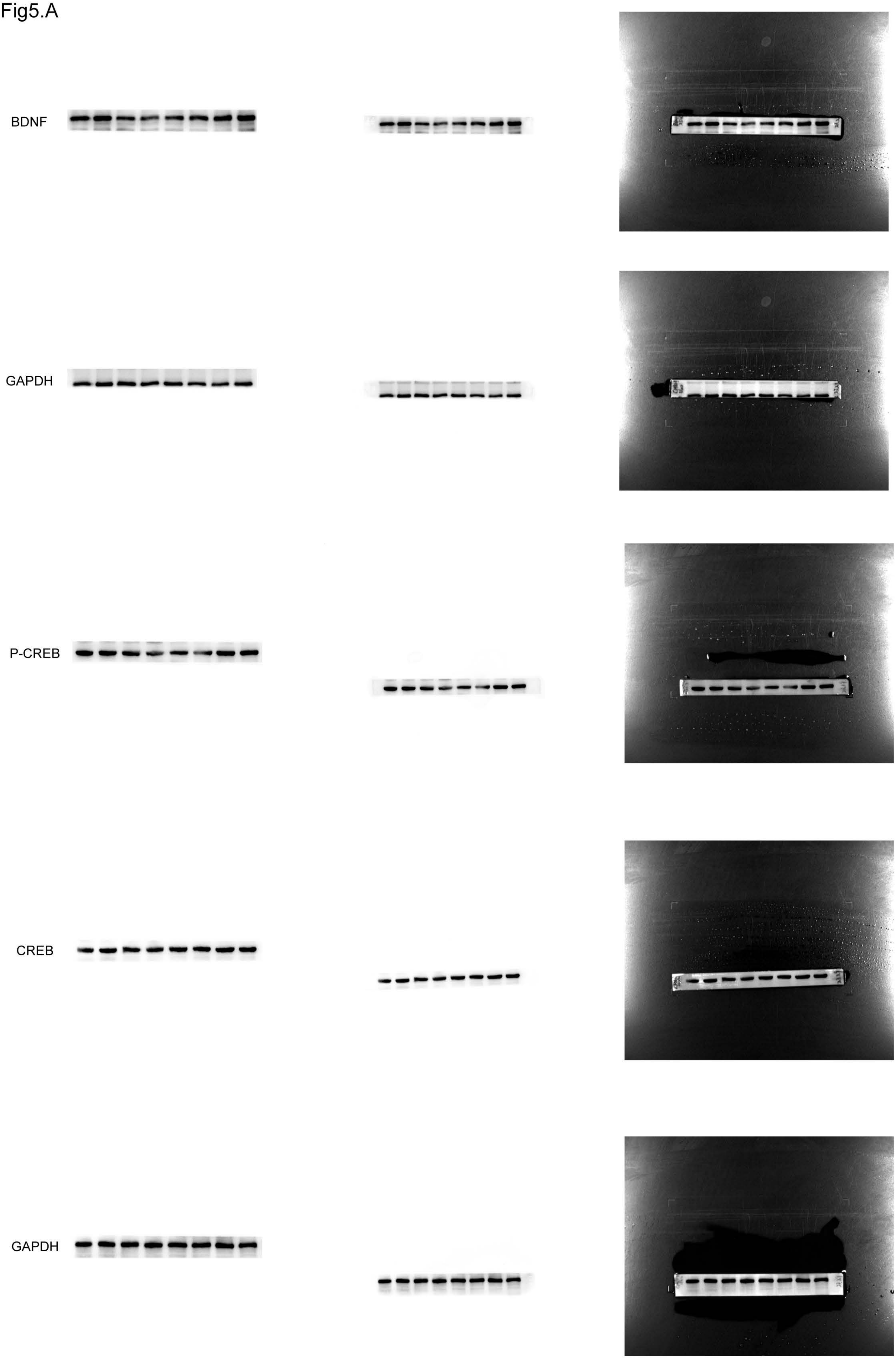

Fig5.D

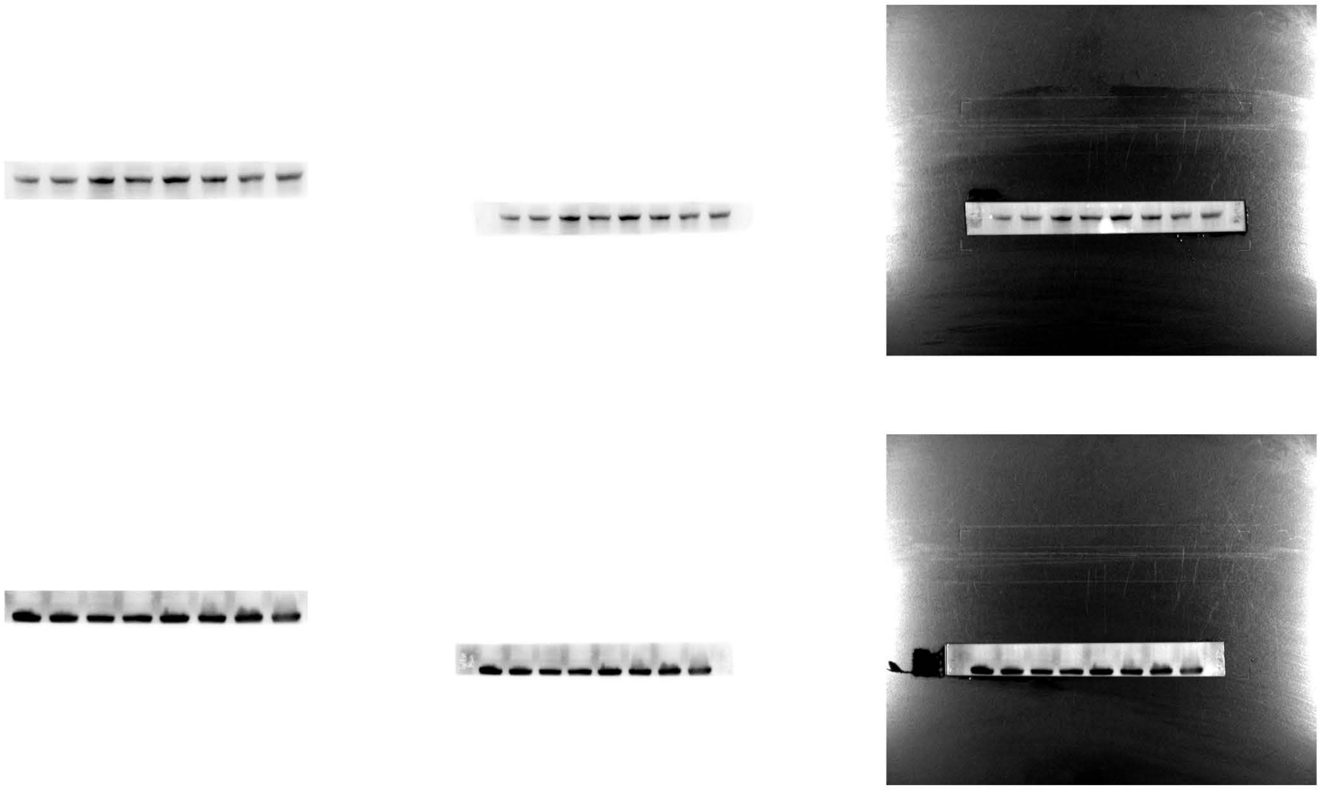

Fig6.A

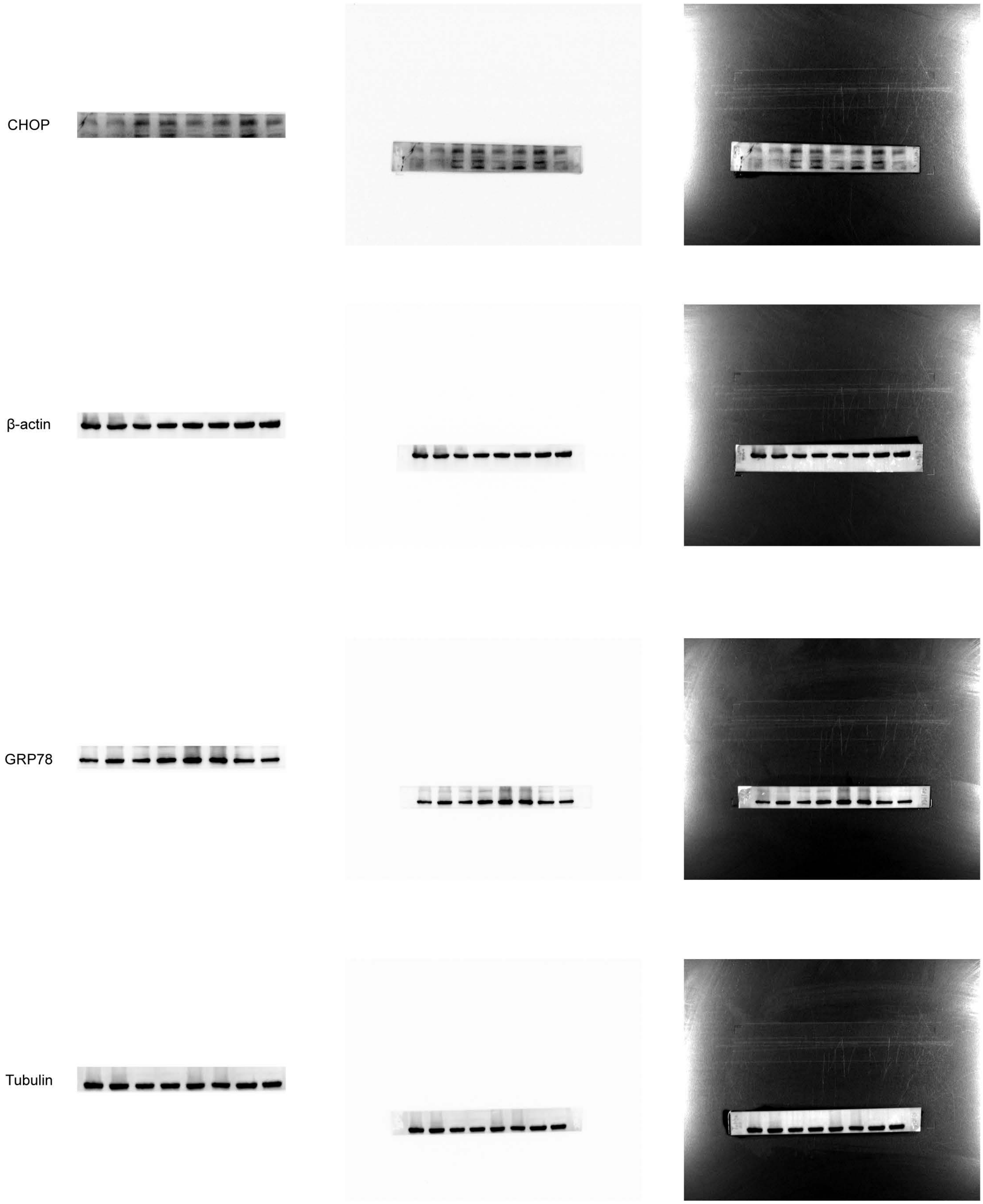

Fig6.B

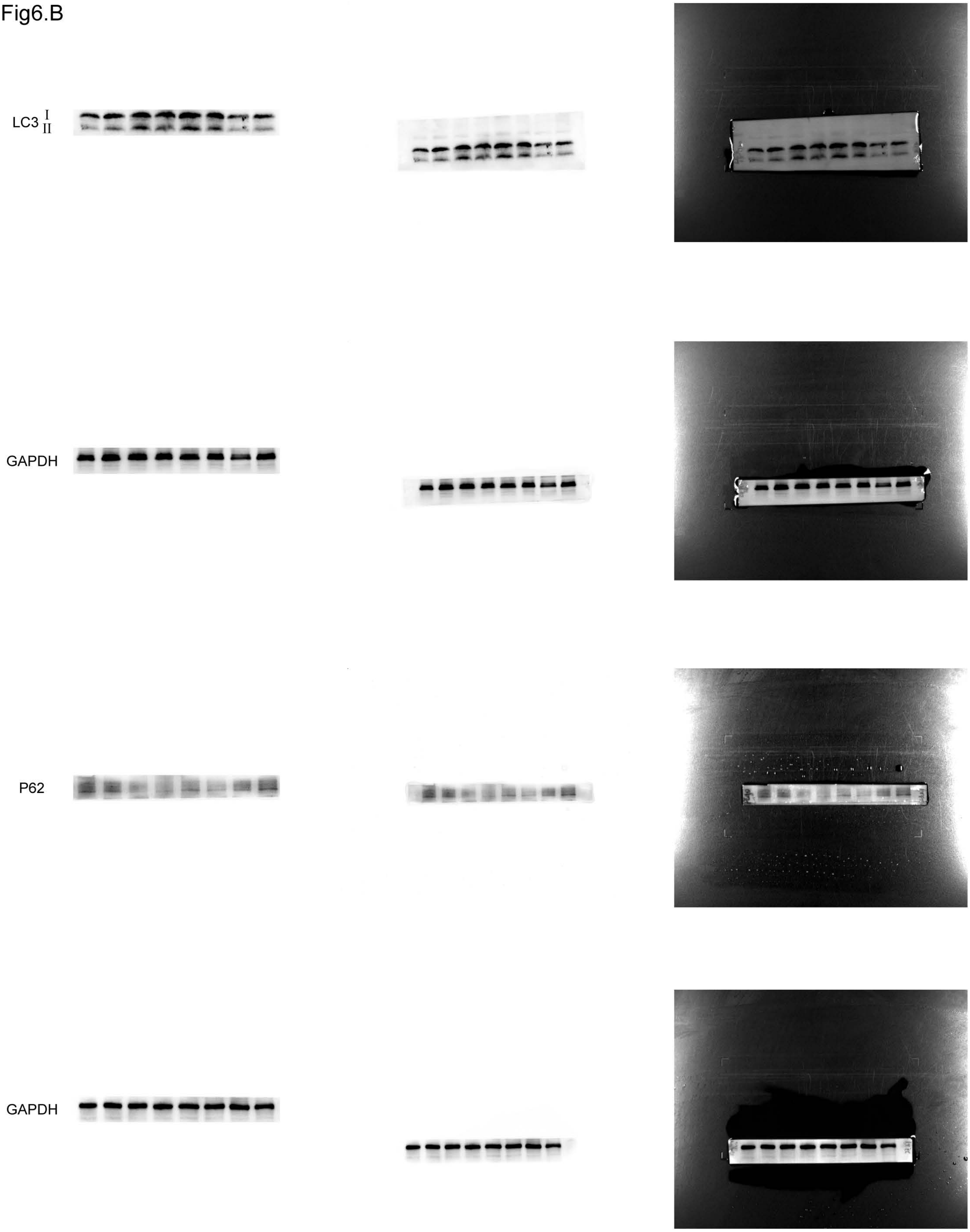

Fig6.B

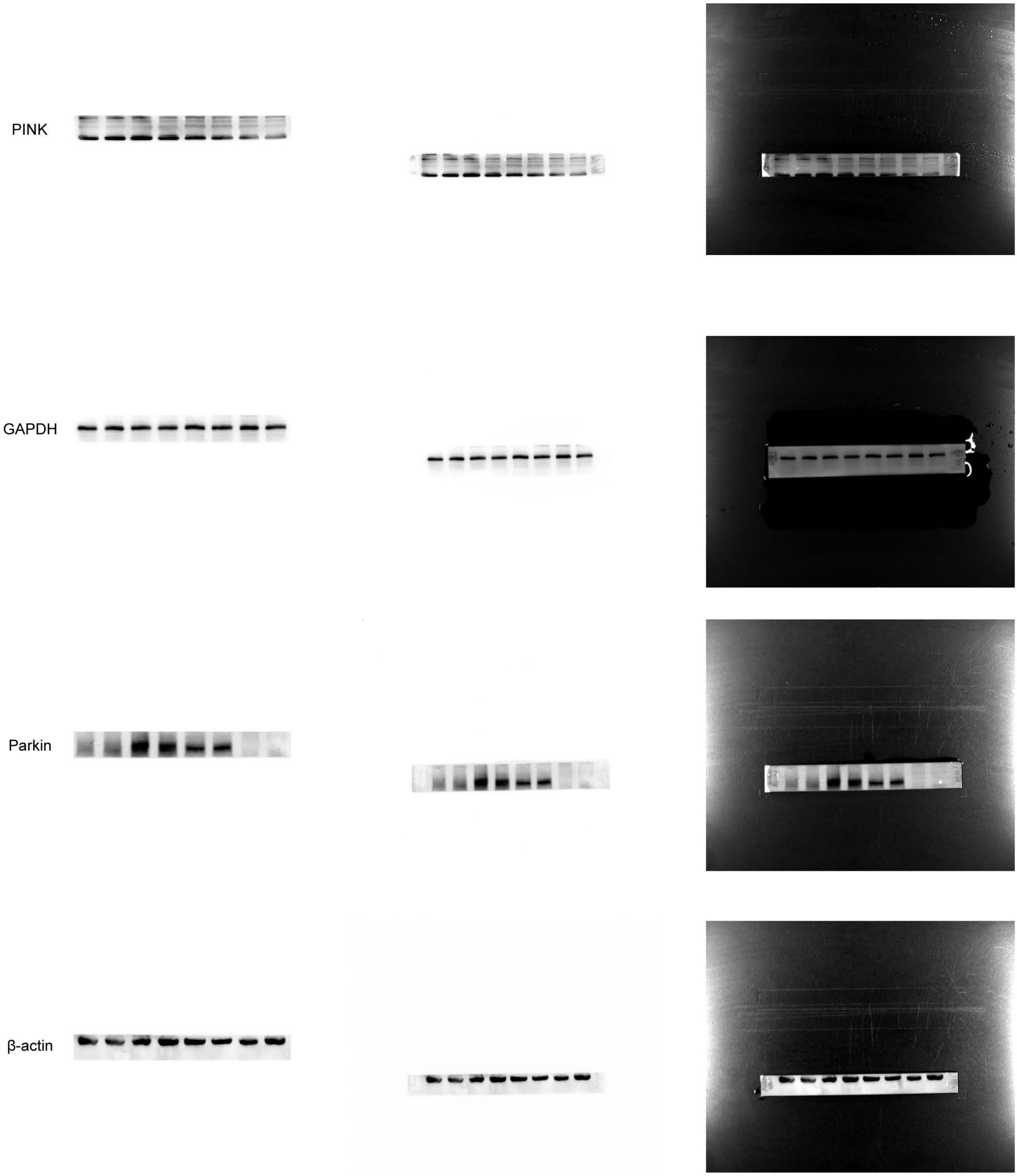

Fig8.D

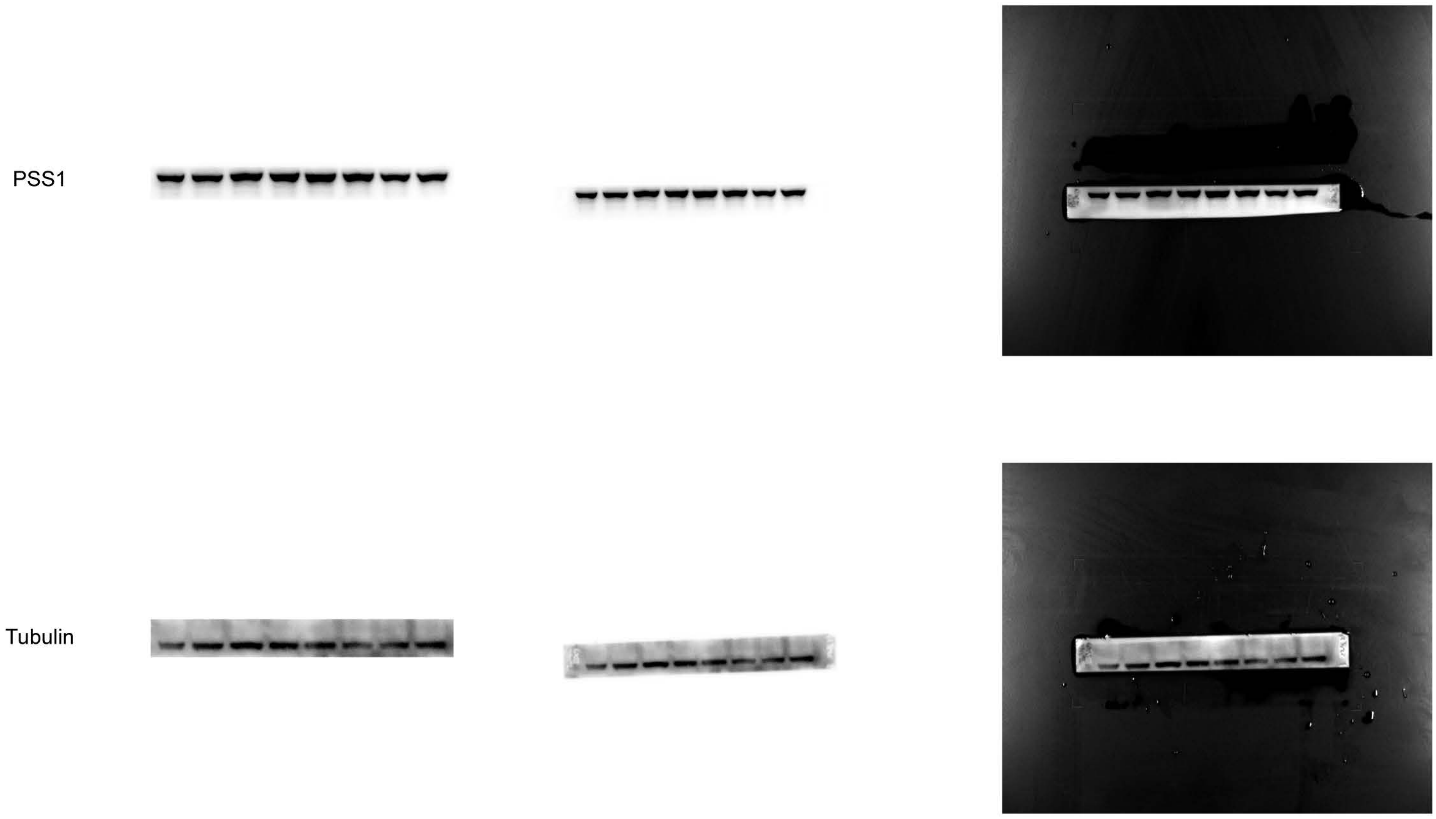

Fig8.E

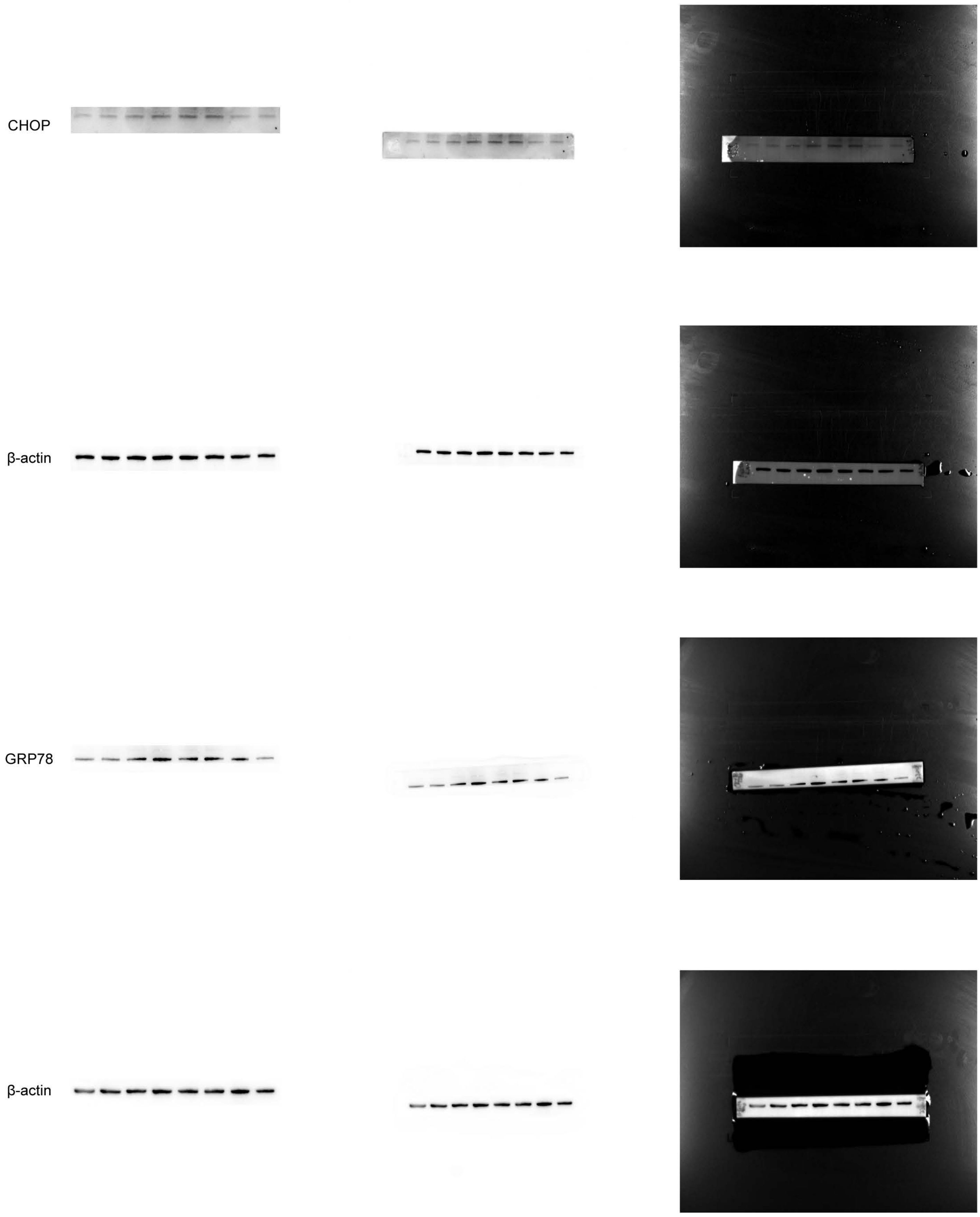

Fig8.F

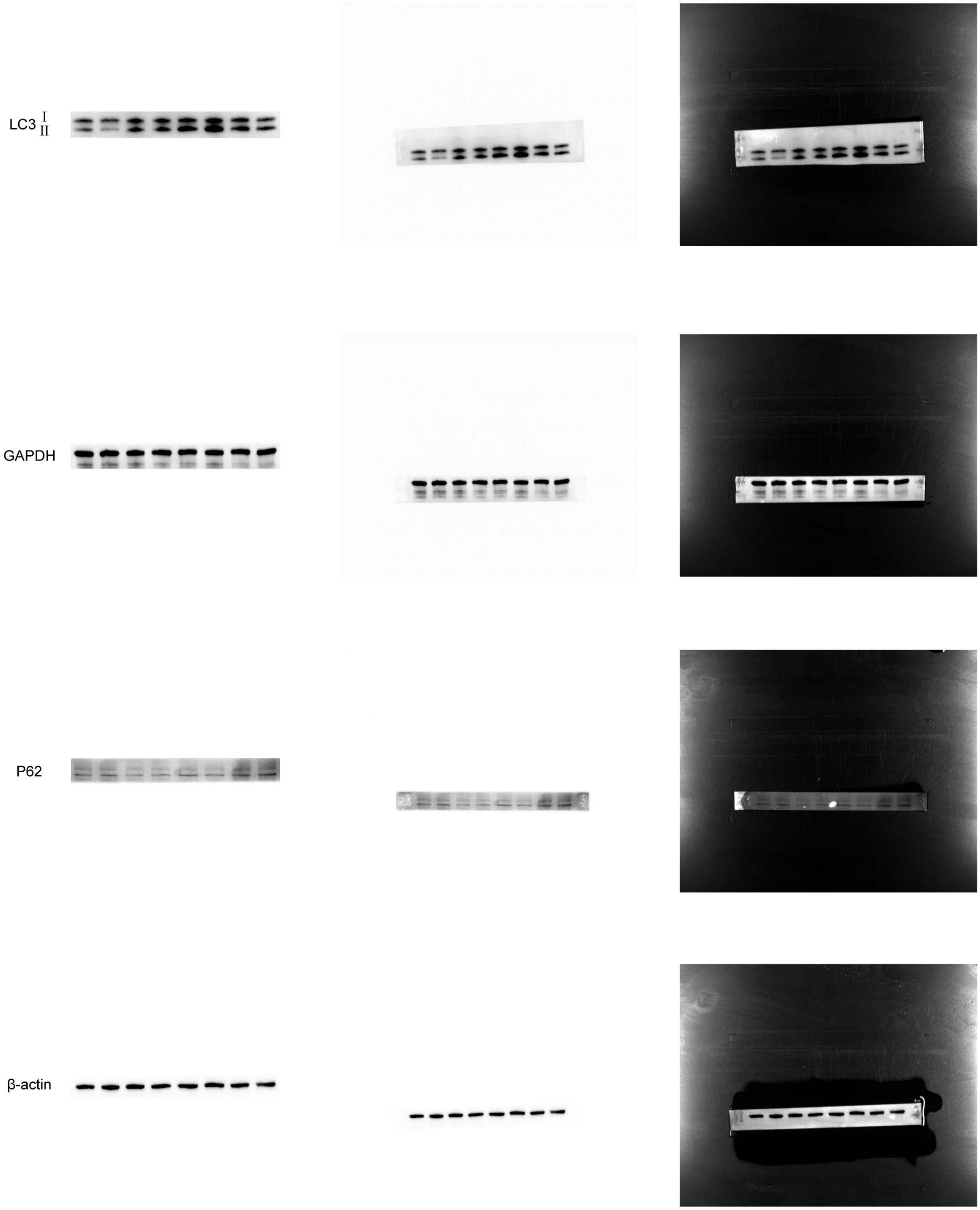

Fig8.F

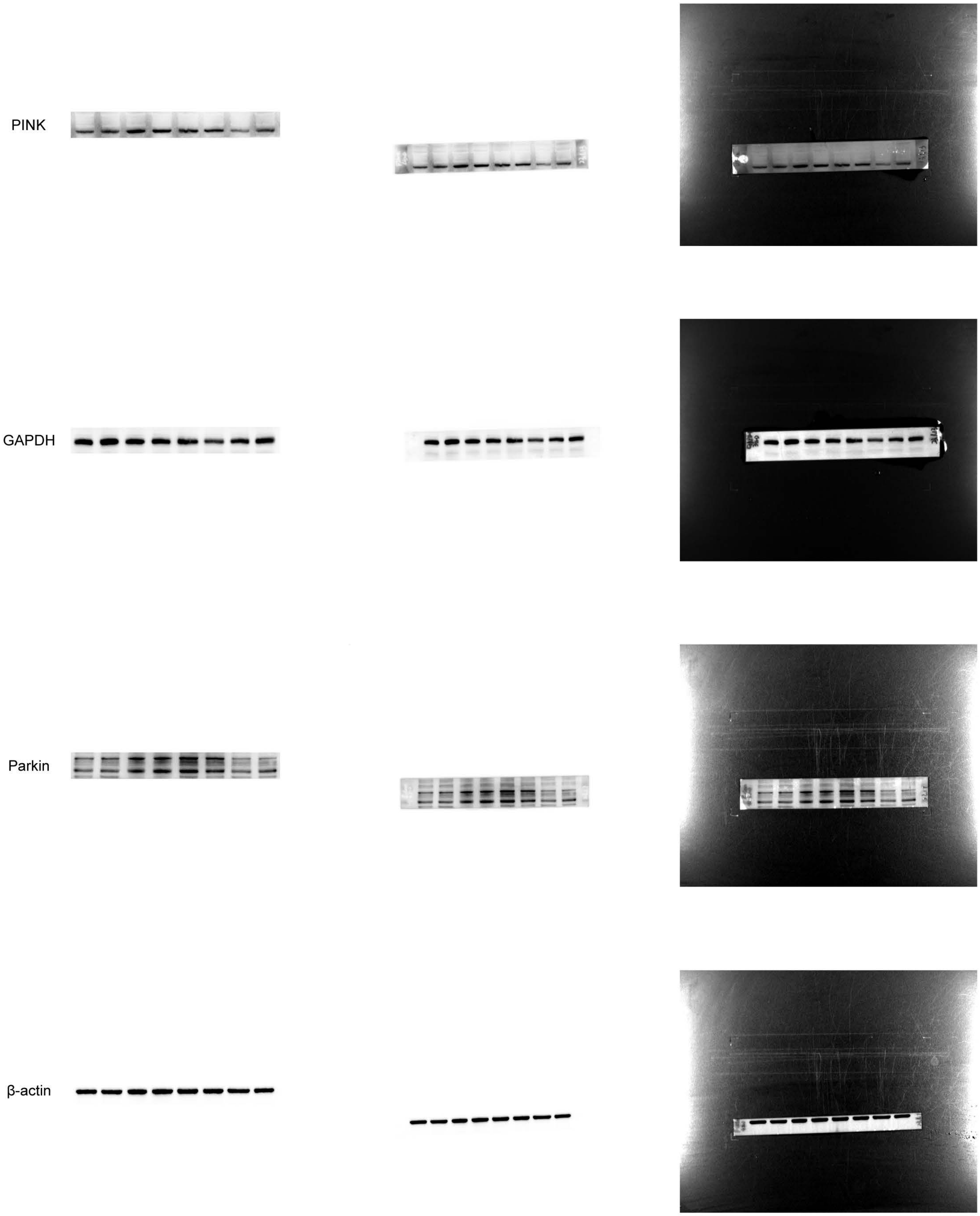

Additional file 5
Fig.S4.B

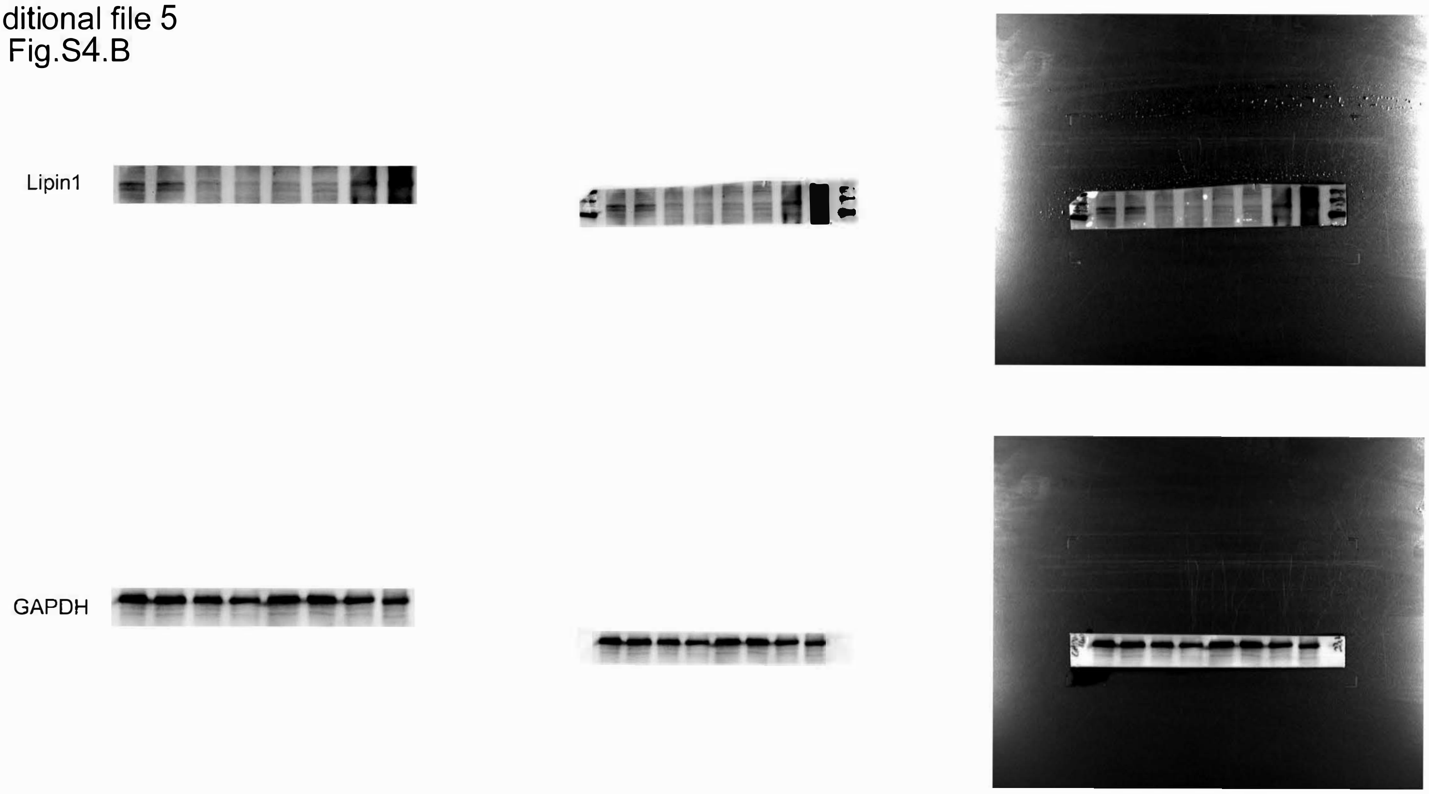

Additional file 8
Fig.S6.B

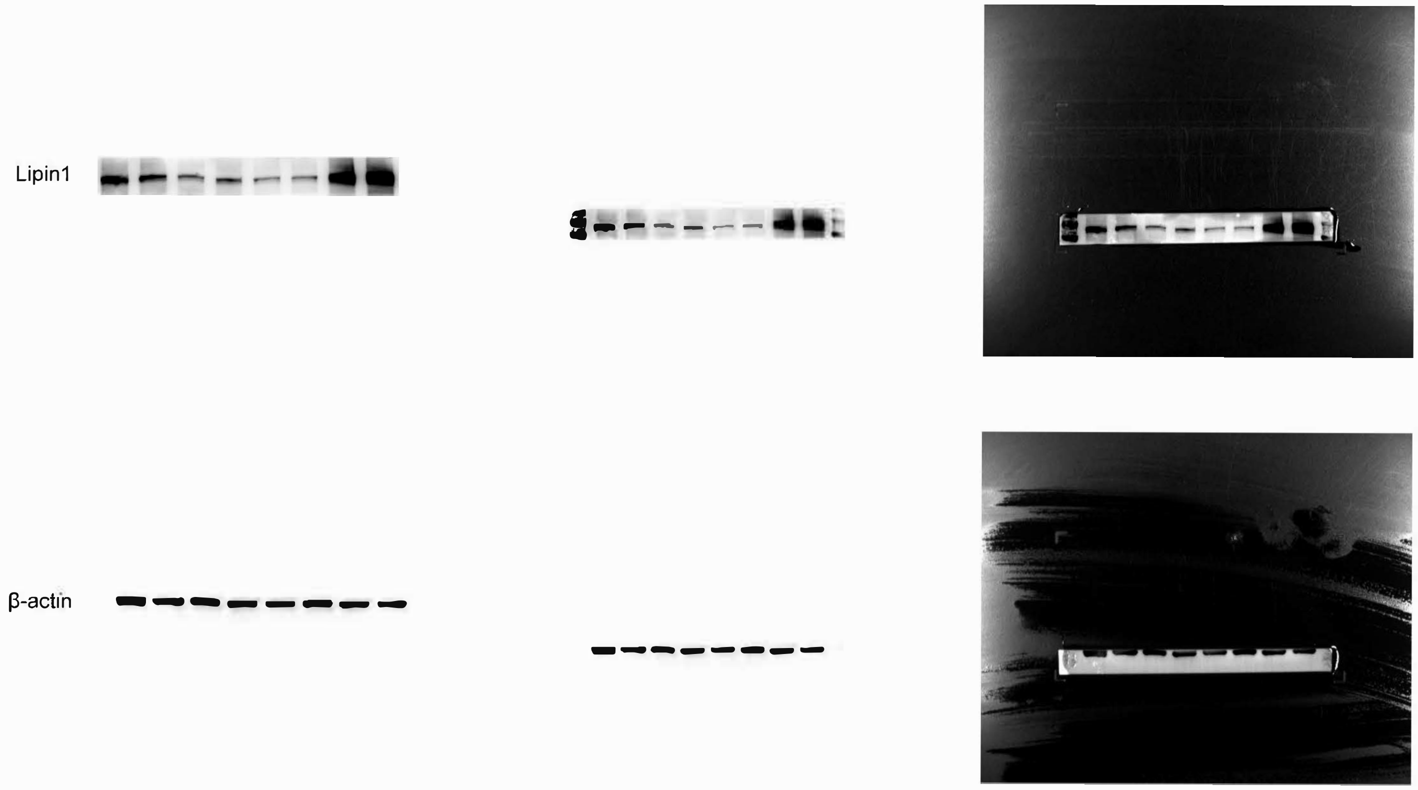

Supplement: Supplementary file 9 — Supplementary Material 9 [file 12974_2025_3441_MOESM9_ESM.pdf]
